# Changes in the Morphological Diversity of Larvae of Lance Lacewings, Mantis Lacewings and Their Closer Relatives over 100 Million Years

**DOI:** 10.3390/insects12100860

**Published:** 2021-09-23

**Authors:** Joachim T. Haug, Gideon T. Haug, Ana Zippel, Serita van der Wal, Patrick Müller, Carsten Gröhn, Jörg Wunderlich, Christel Hoffeins, Hans-Werner Hoffeins, Carolin Haug

**Affiliations:** 1 Biocenter, Ludwig-Maximilians-Universität München (LMU Munich), Großhaderner Str. 2, 82152 Planegg-Martinsried, Germany; jhaug@bio.lmu.de (J.T.H.); gideon.haug@palaeo-evo-devo.info (G.T.H.); zippel@biologie.uni-muenchen.de (A.Z.); vanderwal@biologie.uni-muenchen.de (S.v.d.W.); 2 GeoBio-Center at LMU, Richard-Wagner-Str. 10, 80333 München, Germany; 3 Independent Researcher, Kreuzbergstr. 90, 66482 Zweibrücken, Germany; pat14789@web.de; 4 Independent Researcher, Bünebüttler Weg 7, 21509 Glinde, Germany; jcgroehn@t-online.de; 5 Independent Researcher, Oberer Haeuselbergweg 24, 69493 Hirschberg, Germany; joergwunderlich@t-online.de; 6 Independent Researcher, Liseistieg 10, 22149 Hamburg, Germany; chw.hoffeins@googlemail.com (C.H.); hoffeins@aol.com (H.-W.H.)

**Keywords:** Neuroptera, Mantispidae, Berothidae, Osmylidae, quantitative morphology, ontogeny

## Abstract

**Simple Summary:**

Among insects, the group Neuroptera (lacewings) appears to be less well known to most people, at least in comparison to beetles, butterflies, or wasps. Nowadays, about 6000 species of lacewings are known, but the fossil record yields a large number of further representatives, hinting to a possibly larger role lacewings played in ecosystems of the past. Especially the larvae of lacewings (most prominent examples are antlions) exhibit a large morphological diversity, with fossils exhibiting many peculiar morphologies not known from the modern fauna. Lacewing larvae are well recognisable as such by their prominent jaws, also called stylets. In this study, we analysed the change of larval diversity in different lacewing groups based on the shape of these stylets together with the heads. Our results point to a complex pattern of diversity changes in Neuroptera from 100 million years ago until today.

**Abstract:**

Neuroptera, the group of lacewings, comprises only about 6000 species in the modern fauna, but is generally assumed to have been more diverse and important in the past. A major factor of the modern-day ecological diversity of the group, and supposedly in the past as well, is represented by the highly specialised larval forms of lacewings. Quantitative analyses of the morphology of larvae revealed a loss of morphological diversity in several lineages. Here we explored the diversity of the larvae of mantis lacewings (Mantispidae), lance lacewings (Osmylidae), beaded lacewings (Berothidae and Rhachiberothidae, the latter potentially an ingroup of Berothidae), and pleasing lacewings (Dilaridae), as well as fossil larvae, preserved in amber, resembling these. We used shape analysis of the head capsule and stylets (pair of conjoined jaws) as a basis due to the high availability of this body region in extant and fossil specimens and the ecological importance of this region. The analysis revealed a rather constant morphological diversity in Berothidae. Mantispidae appears to have lost certain forms of larvae, but has seen a drastic increase of larval diversity after the Cretaceous; this is in contrast to a significant decrease in diversity in adult forms.

## 1. Introduction

Lacewings seem to be lesser-known representatives of the group Insecta, at least in comparison with butterflies, beetles, or wasps. In fact, many people may see lacewings regularly, but not recognise them as such, as many winged lacewings resemble butterflies, dragonflies, or even praying mantises at first glance [1,2,3]. The group of lacewings (Neuroptera) is an ingroup of Holometabola, and as other representatives of this group (such as beetles, butterflies, flies, wasps) which are also representatives of Neuroptera have highly specialised larvae that differ significantly from the corresponding adult (for challenges with the term larva, especially in the group Holometabola, see [4]). The specialisation of larvae of lacewings is coupled to their lifestyle. They are mostly highly specialised predators; their upper jaws form a pair of venom-injecting sucking stylets with their corresponding lower jaws [5].

As in many piercing-sucking mouthparts, those of lacewing larvae also face a significant mechanical challenge: to penetrate the surface of a prey item it is necessary to produce a counteracting force, otherwise the predator pushes itself back when attempting to pierce the prey, or just pushes the prey away from itself. The solution for this challenge in most lacewing larvae is simple: counteracting mouthparts. The paired stylets can be curved so that the tips face each other. When such stylets snap together with the prey between them, the force produced by one stylet counteracts that of the other one, leaving no resulting force pushing back the predator, instead transmitting this force onto the prey surface, thus easily penetrating it.

It may therefore not be surprising that many lacewings possess strongly curved stylets, as do the larvae of antlions (Myrmeleontidae), owl “flies” (should better be termed owl lacewings for consistency; Ascalaphidae), spoon-winged lacewings (Nemopterinae), split-footed lacewings (Nymphidae), long-necked antlions (larvae of thread-winged lacewings; Crocinae), long-nosed antlions (larvae of silky lacewings; Psychopsidae), aphid lions (larvae of green and brown lacewings; Chrysopidae and Hemerobiidae), and even the aquatic larvae of Nevrorthidae (no vernacular name for representatives of this rather species-poor group). Yet, in the latter, the stylets are slightly special in being rather straight proximally, but strongly curved distally [6].

Still, in some lineages of Neuroptera, straight stylets have evolved in the larvae. It seems quite possible that curved stylets are the ground pattern condition and that straight stylets evolved repetitively [7], yet how often exactly straight stylets evolved remains unclear. Straight stylets are clearly present in larvae of dusty lacewings (Coniopterygidae), beaded lacewings (Berothidae and Rhachiberothidae, the latter potentially ingroup of Berothidae), and pleasing lacewings (Dilaridae). Furthermore, there are special conditions in several lineages. Many larvae of mantis lacewings (Mantispidae) have straight stylets, but others have slightly curved ones, although much broader and less curved than, for example, in aphid lions. Larvae of spongilla “flies” (should be termed spongilla lacewings for consistency; Sisyridae) have very long and thin stylets. They are, in fact, so thin that they become flexible to a certain degree and can appear curved or rather bent [8,9]. Larvae of lance lacewings (Osmylidae) have stylets that are principally straight, but slightly outward curving. Finally, there is one very unusual fossil larva with very long stylets which are slightly S-curved, but functionally straight as well [7].

Larvae with straight stylets often have very distinct ecologies. Many larval lance lacewings are semi-aquatic predators, the only lacewings with this lifestyle [10]. Larvae of mantis lacewings interact with spiders [11,12], those of beaded lacewings with termites (e.g., [13] and references therein), and those of spongilla lacewings with freshwater sponges [14]. It therefore appears that the specialisation of the straight mouthparts in lacewing larvae is often coupled to very distinct ecological roles.

We still (only) attempt to understand the modern-day ecosystems and how they change over time. Looking back in time into fossil ecosystems has the potential to improve our understanding about ecosystem changes over longer time periods. Still, larval forms are often not involved when it comes to such reconstructions, although especially the larvae of holometabolans (hence including lacewings) are well known to fulfil many important ecosystem functions (possibly even more so than the adults). This situation may appear counter-intuitive at first glance, yet many reconstructions of past ecosystems rely on taxonomic information, and larvae are more challenging to interpret from a taxonomic point of view. Ecological interpretations also rely on a comparison to modern forms, and often the ecology of adults is better known than that of larvae.

Still, the very distinct morphologies of lacewing larvae indeed provide some indication on their ecology. Haug et al. [15,16,17] argued that morphological diversity of larvae can act as a proxy for their ecological diversity by using quantitative shape analysis. Such methods have also been used successfully for identifying similarities and differences in lacewing larvae [7,18,19,20].

We here compiled all known reports of a distinct subset of extant and fossil lacewing larvae with (more or less) straight stylets. We reported additional specimens and compared the morphological diversity of such larvae in the fossil record and in modern ecosystems with outline analysis via elliptic Fourier transformation.

## 2. Material and Methods

### 2.1. Material

Material for this study came from various sources. Many specimens, especially from the extant fauna, were investigated via depictions in the literature. We performed an intense literature search via standard literature databases and included both backward and forward reference searching to achieve an as complete dataset as possible. In this way, we considered all available depictions of larvae of the groups Dilaridae, Berothidae, Rhachiberothidae (possibly an ingroup of the former one), Mantispidae, and Osmylidae. All of these larvae are characterised by prominent straight or almost straight stylets.

We considered all these larvae in one analysis as we aimed to compare them to fossil larvae (see further below). It is not always simple to identify which of these lineages the fossil larvae are representatives of (see, e.g., discussion in [21]). Analysing all of these together reduces the effects of misidentification that can occur when analysing these groups separately (for example, a larva of Osmylidae interpreted as a larva of Dilaridae). Additionally, if the extant forms potentially show a separation in the analysis, this might provide additional cues for identifying the fossil larvae.

We did not include larvae of Sisyridae in this analysis. Even though larvae of Sisyridae have stylets that are principally straight, these stylets are so slender that they are flexible. This makes them very different from the mouthparts of the other larvae and would heavily polarise the analysis, possibly concealing differences of the other groups by compressing the variation among them into a smaller region of the explored space (see below for more details).

For the same reason, we did not include larvae of the group Coniopterygidae. In most larvae the stylets are rather small and indistinct, often not visible under the strongly protruding labrum [1] (his Figure 29.9) [22] (his Figure N2I,J) [23] (his Figures 62–65, *Coniopteryx vicin*) [24] (his Figure 6, *Helicoconis* sp.) [25] (his Figure 135, *Conwentzia barretti*) [26] (their Figures 6–15, *Semidalis pluriramosa* and *S. pseudouncinata*) [27] (their Figures 1–4, *Conwentzia pineticola* and *C. psociformis*). In cases in which they are not concealed by the labrum, they remain very slender ([23] (his Figures 58 and 59 pl. XIX, ?*Helicoconis lutea*) [28] (his Figures 8 and 9, *Aleuropteryx lutea*) [29] (his Figure 2 p. 76, *Aleuropteryx juniperi*)). The overall body shape is also very distinct from that of other lacewing larvae, making it easier to identify them as such.

Additionally, many fossil larvae with straight stylets were investigated via depictions in the literature. In addition, we directly documented specimens from different collections and preserved in different ambers, namely in Eocene Baltic amber (ca. 40 million years old) and Cretaceous Kachin amber from Myanmar (ca. 99 million years old) [30,31,32]. Specimen CCHH 1270-1 was preserved in Baltic amber and from the collection of two of the authors (CH and HWH, to be deposited in the amber collection at the Senckenberg Deutsches Entomologisches Institut Müncheberg; SDEI Lep-103564). Specimen SMF Be 1297 was also preserved in Baltic Amber and from the Senckenberg Forschungsinstitut und Naturmuseum in Frankfurt/Main. Specimen Gröhn 7512 was also preserved in Baltic Amber and from the collection of one of the authors (CG). Specimens BUB 0049, 3064, 3065, 3144, 3348, 3355, 3368, 3390, 3726, 3737, 3741, 3962, and 3963 were all preserved in Kachin amber from Myanmar and are from the collection of one of the authors (PM). Specimens CJW F 3197, 3198, and 3336 were also preserved in Kachin amber and are from the collection of one of the authors (JW). Specimens PED 0380, 0587, 0627, 0769, 0772, 0790, 0791, 0823, 0828, 0898, and 0900 were also preserved in Kachin amber and are from the Palaeo-Evo-Devo Research Group Collection of Arthropods, Ludwig-Maximilians-Universität München. The specimens from the PED collection were legally purchased on ebay.com from various traders (macro-cretaceous, burmite-miner, burmite-researcher).

### 2.2. Documentation and Image Processing

Documentation of the specimens in amber was performed on a Keyence VHX 6000 microscope. We photographed each specimen from two sides (if accessible), illuminated by coaxial cross-polarised light [33] as well as with unpolarised ring light. Under both illuminations, specimens were documented once with a white background and once with a black background. The images providing the best contrast were used.

Images were processed automatically by the built-in software of the microscope. Adobe Photoshop CS2 and CS3 were used for optimising all images (histograms, saturation, sharpness). All visible structures of the fossils were colour marked to provide the reader with an interpretation of all accessible structures.

### 2.3. Shape Analysis

A comparative statistical analysis of the morphology of the specimens was conducted by a Principal Component Analysis (PCA) of the results of an Elliptic Fourier analysis. All accessible heads (head capsules) were redrawn by hand in Adobe Illustrator CS2. Hereby, the better-preserved half of the head was drawn and mirrored. The resulting drawing was checked against the original image to reduce possible artefacts. Dorsal and ventral views were used even though there are slight differences, but the important criterion was a well-accessible posterior rim of the head capsule.

Redrawn images were analysed in SHAPE (© National Agricultural Research Organization of Japan), a free software providing the tools to perform Elliptic Fourier analysis and PCA. The software transforms the outlines of the reconstructed head drawings into a vectorised object, called a chain code. To achieve this, the program uses a vector-based step-by-step approximation of an ellipse to the outline of the head. The vectorised shapes (chain codes) are represented by numeric values, which are then transformed into normalised Elliptic Fourier Descriptors (EFDs). This method represents a variation of the well-known Fourier transformation, practically applied on shapes of natural objects. The EFDs were finally analysed with a PCA that resulted in the most important characters for morphological diversity in the data set. The entire procedure including the PCA was applied following [34,35]. The results of the PCA were visualised using OpenOffice Calc and redrawn in Adobe Illustrator CS2 without changing the position of the data points. 

## 3. Results

All occurrences of larvae were listed chronologically. Cases in which the same specimen had been re-figured were also included chronologically with reference to the original occurrence. While this includes a certain redundancy, it should represent the most complete way of cross-referencing, avoiding interpreting the same specimens as two independent occurrences (this way of presentation has also been used for other ingroups of Neuroptera [6,15,16,17]). For an easier cross-reference to our dataset, we provide the dataset number here along with the specimen. The dataset number is a four-digit number; the first two numbers provide a reference to the ingroup of Neuroptera, and the last two represent a simple chronological numeration.

### 3.1. Extant Larvae of Berothidae

In total, twelve extant specimens of larvae of Berothidae (specimens 5001–5012) were found in the literature that could be used for our analysis. These were presented in 20 publications, which are listed in the following.

(1) Tillyard [36] depicted a drawing of a stage 1 larva (hatchling) of *Spermophorella disseminata* (specimen 5001) in dorsal view (his Figure 32 pl. XVIII). A close-up of the head was provided, also in dorsal view (his Figure 33 pl. XVIII). The size of the specimen was provided as a magnification factor, which is not informative in combination with the electronic versions available to the present authors. The specimen was re-figured by Tillyard [37], Gurney [38], and Tjeder [39].
(2) Tillyard [37] re-figured specimen 5001 (his Figure U8D p. 316), i.e., the drawing from Tillyard [36].
(3) Gurney [38] depicted several drawings of larvae of Berothidae. The first specimen was a stage 1 larva of Lomamyia sp. (specimen 5002) in dorsal view (his Figure 2 pl. 11 p. 163). The length was stated as 2.04 mm. Additionally, a detail of the head of a stage 1 larva was provided (his Figure 8 pl. 12 p. 165), as well as a detail of the maxilla (his Figure 7 pl. 12 p. 165). It remains unclear whether it is the same specimen, but we assume that to be the case, not considering the head as a separate specimen. The specimen was re-figured by Tjeder [39].
  The second specimen was a stage 3 larva (“mature larva”) of *Lomamyia* sp. (specimen 5003) in dorsal view (his Figure 5 pl. 11 p. 163). The length was stated as 9.36 mm. Additionally, a detail of the head of the specimen was provided in dorsal view (his Figure 12 pl. 12 p. 165) and ventral view (his Figure 13 pl. 13 p. 167), as well as details of the trunk appendages (his Figures 18 and 22 pl. 14 p. 169). The specimen was re-figured by Tjeder [39] and Tauber [40].
  Gurney also re-figured specimen 5001 (his Figure 4 pl. 11 p. 163), i.e., the drawing from Tillyard [36], including the detail of the head region (his Figure 10 pl. 12 p. 165).
(4) Tjeder [39] summarised and re-figured the known larvae of the group Berothidae (all on p. 259). This includes drawings of specimen 5001 (habitus and head; his Figures 212 and 213), i.e., drawings from Tillyard [36], and of specimens 5002 and 5003 (habitus and head; his Figures 214–218), i.e., drawings from Gurney [38].
(5) MacLeod [23] depicted a drawing of a stage 3 larva (specimen 5004) of *Lomamyia flavicornis* in dorsal (his Figure 36 pl. XII) and ventral view (his Figure 37 pl. XII), as well as a detail of the mouth region (his Figure 38 pl. XII). According to the scale provided, the head capsule measured 0.37 mm.
(6) Toschi [41] depicted the drawing of a head of a stage 1 larva (specimen 5005) of *Lomamyia latipennis* in dorsal view (her Figure 3 p. 25). According to the text, she had several specimens available. The drawing appears to be based on an actual specimen. No indication of size was provided.
(7) Tauber and Tauber [42] depicted micrographs of stage 2 and 3 larvae (their Figures 1–3 p. 626) of Lomamyia lattipennis. A drawing of a head in dorsal view (their Figure 4 p. 628) was also provided. Yet, the micrographs were recorded on moving animals and do not allow the recognition of the outline of the head. The drawing is incomplete, not providing the distal region of the stylets. Therefore, we cannot consider any of the specimens for our analysis.
(8) Riek [1] depicted the drawing of a head of a larva of unknown stage and species (“berothid”) in ventral view (his Figure 29.5B p. 476). He also provided the habitus in dorsal view (his Figure 29.10H p. 486), as well as a dorsal view of the head (his Figure 29.10G p. 486). All depictions match exactly and are therefore interpreted as original, based on a single specimen (specimen 5006). No indication of size was provided. The specimen was re-figured by Gepp [24], New [43], and Tauber et al. [44].
(9) Gepp [24] re-figured specimen 5006, the specimen from Riek [1], more precisely the habitus in dorsal view (his Figure 8a pl. 4 p. 195) and the dorsal head detail (his Figure 8b pl. 4 p. 195).
(10) Brushwein [45] depicted several immature stages of *Lomamyia hamata*. Yet, none of the images provide details of the head in dorsal view. Hence, none of the specimens could be further considered in our study.
(11) Tauber [40] re-figured the habitus of specimen 5003 (her Figure 33.18 p. 134), i.e., a specimen from Gurney [38], as well as the drawing of the head (her Figure 33.19 p. 134) from Tauber and Tauber [42]. There are later editions of this contribution also figuring this image (at least 1987, 1991, 2008, our version was labelled 1991). We assume that all of these contributions feature the same images. Hence, these refer to the same specimen.
(12) Minter [46] depicted two larval specimens of Berothidae and Rhachiberothidae (considered to be ingroup of Berothidae by several authors, e.g., [13]). The first (specimen 5007) was a drawing in dorsal view of a stage 1 larva of Mucroberotha vesicaria (his Figure 5 p. 118). In addition, he provided drawings of the head in dorsal (his Figure 6 p. 118) and ventral view (his Figure 7 p. 118). Furthermore, micrographs of the eye region (his Figure 8 p. 119) and the empodium (his Figure 9 p. 119) were provided. According to the text, the drawings are based on an actual specimen collected in South Africa. According to the provided scale, the specimen was 1.01 mm long.
  The second specimen (specimen 5008) was a drawing in dorsal view of a stage 1 larva of Podaella sp. (his Figure 14 p. 121). In addition, he provided drawings of the head in dorsal (his Figure 15 p. 121) and ventral view (his Figure 16 p. 121). Furthermore, micrographs of the antero-lateral head region (his Figure 17 p. 122) and the empodium (his Figure 18 p. 122) were provided. According to the text, the drawing is also based on an actual specimen collected in South Africa. According to the provided scale, the specimen was 1.13 mm long.
(13) New [43] re-figured specimen 5006, i.e., the specimen from Riek [1], with two images, head and habitus, both in dorsal view (his Figure 34.10A,B). The source was not directly seen by the current authors, but is based on information from Tauber et al. [44] and Wedmann et al. [13].
(14) Tauber et al. [44] re-figured specimen 5006, i.e., the specimen from Riek [1] (but citing New [43] as source), with two images: head and habitus, both in dorsal view (their Figure 6K,L p. 793).
(15) Möller [47] reported on larvae of *Podallea vasseana.* The work is a master’s thesis and was not available to the present authors (cited after [13]). We assume that the specimens used were the same as in Möller et al. [48] (see next point).
(16) Möller et al. [48] provided images of several larvae of Berothidae. The first (specimen 5009) is a stage 1 larva of Podallea vasseana. Images include drawings of the entire larva in dorsal view (his Figure 7 p. 5), head in dorsal (his Figure 8 p. 5) and ventral view (his Figure 9 p. 5), trunk appendages (his Figure 10 p. 5), and details of the setae arrangement (chaetotaxy) of the trunk segments (his Figures 11–15 p. 5). Additionally, several details were provided as SEM micrographs, including details of the antennae (his Figures 16 and 17 p. 6), tip of a stylet (his Figure 18 p. 6), setae on the maxilla (his Figure 19 p. 6), details of setae (his Figure 20 p. 6), and the anus region (his Figure 21 p. 6). According to the text, the drawings were based on an actual specimen collected in South Africa. According to the provided scale, the specimen was 1.25 mm long.
  The second specimen is a stage 2 larva of *Podallea vasseana*. Yet, none of the images provide access to the stylets in relation to the head capsule, hence the specimen could not be further considered here.
  The third (specimen 5010) is a stage 3 larva of Podallea vasseana. Images include drawings of the entire larva in dorsal view (his Figure 28 p. 9), head in dorsal (his Figure 29 p. 9) and ventral view (his Figure 30 p. 9), trunk appendages (his Figure 36 p. 9), and details of the seta arrangement (chaetotaxy) of the trunk segments (his Figures 31–35 p. 9). Additionally, several details were provided as SEM micrographs, including sensilla on labium (his Figures 37–39 p. 10) and antennae (his Figure 40 p. 10). According to the text, the drawings were based on an actual specimen collected in South Africa. According to the provided scale, the specimen was 3 mm long.
(17) Monserrat [49] depicted several drawings of a stage 1 larva (specimen 5011) of Berothimerobius reticulatus (his Figure 11 p. 197). The images include a habitus drawing (without trunk appendages) in dorsal view (his Figure 11a), the head in dorsal view (his Figure 11b), antenna (his Figure 11c), mandible (his Figure 11d), labial palps (his Figure 11e), maxilla (his Figure 11f), and the three trunk appendages (his Figure 11g–i). According to the text, the drawings were based on an actual specimen. According to the provided scale, the specimen was 1.3 mm long.
(18) Aspöck and Aspöck [3] re-figured specimen 5007 (their Figure 95 p. 488), and 5008 (their Figure 96 p. 488), i.e., the two specimens from Minter [46].
(19) Dobosz and Górski [50] presented a poster with SEM images of a stage 1 larva (specimen 5012) of *Nyrma kervilea*. This included a dorsal view, a ventral view and a dorsal close-up of the head, mouthparts, and trunk appendages. While conference posters are usually not considered valid sources, we opted to include the specimen as (1) the poster was available online, (2) the poster has already been cited in another publication [13], and (3) given the overall rarity, every available specimen is considered.
(20) Heckman [51] provided a figure (his Figure 2.26) that states “unidentified larva in the family Berothidae” in the figure legend. Yet, the specimen is cited as originating from Grebennikov [52]. There, the specimen was interpreted as larva of the group Polystoechotidae [52] (his Figures 30–36 p. 415), which is generally interpreted as an ingroup of Ithonidae in newer analyses. The morphology of the larva is also much more compatible with an interpretation of the specimen as a representative of Ithonidae/Polystoechotidae than Berothidae. Therefore, this specimen was not further considered.

### 3.2. Extant Larvae of Mantispidae

In total, 33 specimens of larvae of Mantispidae (specimens 5201–5233) were found in the literature that could be used for our analysis. These were presented in 53 publications, which are listed in the following.

**Figure 1:**
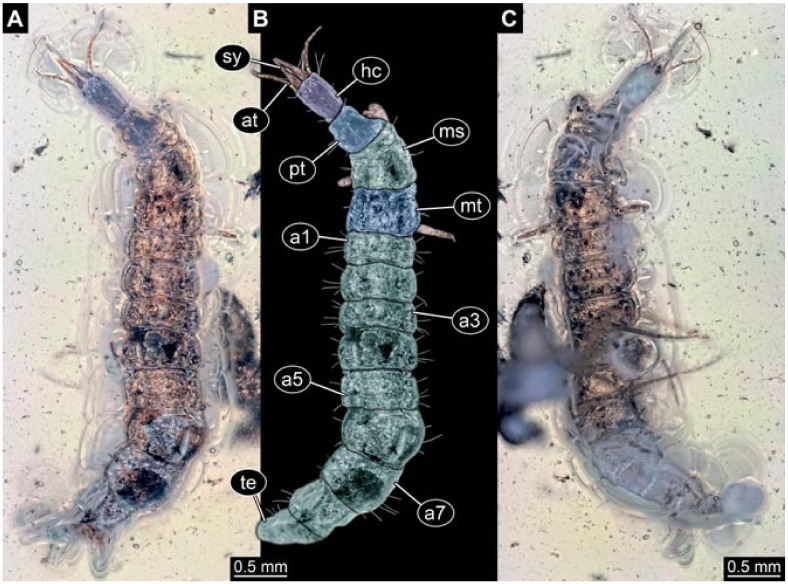
Specimen 5803 (CCHH 1270-1, now SDEI Lep-103564); Baltic amber. (**A**) Dorsal view. (**B**) Dorsal view, colour marked. (**C**) Ventral view. Abbreviations: a1–a7 = abdomen segments 1–7; at = antenna; hc = head capsule; ms = mesothorax; mt = metathorax; pt = prothorax; sy = stylet; te = trunk end.

**Figure 2:**
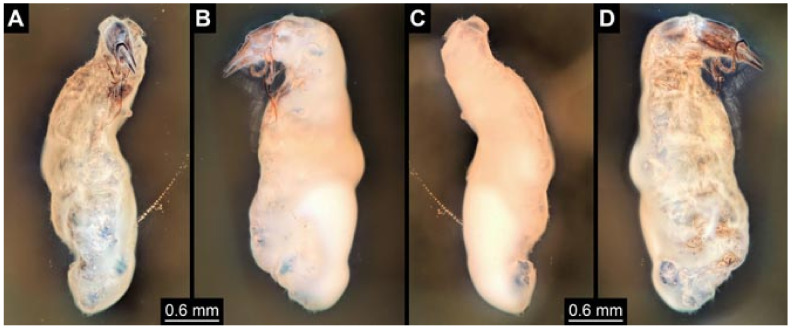
Specimen 5814 (SMF 1297); Baltic amber. (**A**) Ventral view. (**B**) Ventro-lateral view. (**C**) Dorsal view. (**D**) Dorso-lateral view.

**Figure 3:**
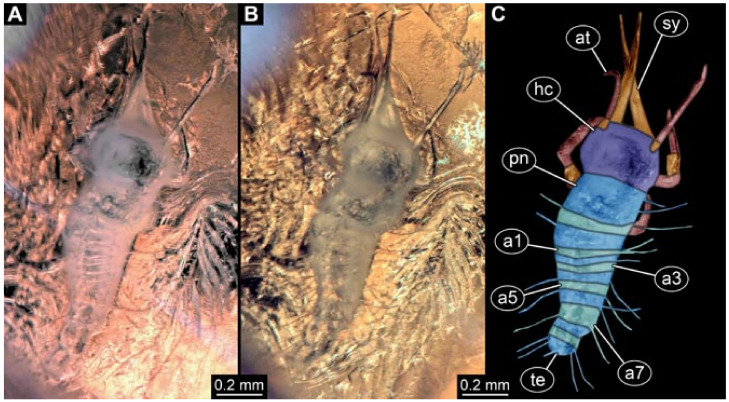
Specimen 5822 (Gröhn 7512); Baltic amber. (**A**) Dorsal view, cross-polarised coaxial light. (**B**) Dorsal view, unpolarised ring light. (**C**) Dorsal view, colour-marked. Abbreviations: a1–a7 = abdomen segments 1–7; at = antenna; hc = head capsule; pn = pronotum; sy = stylet; te = trunk end.

**Figure 4:**
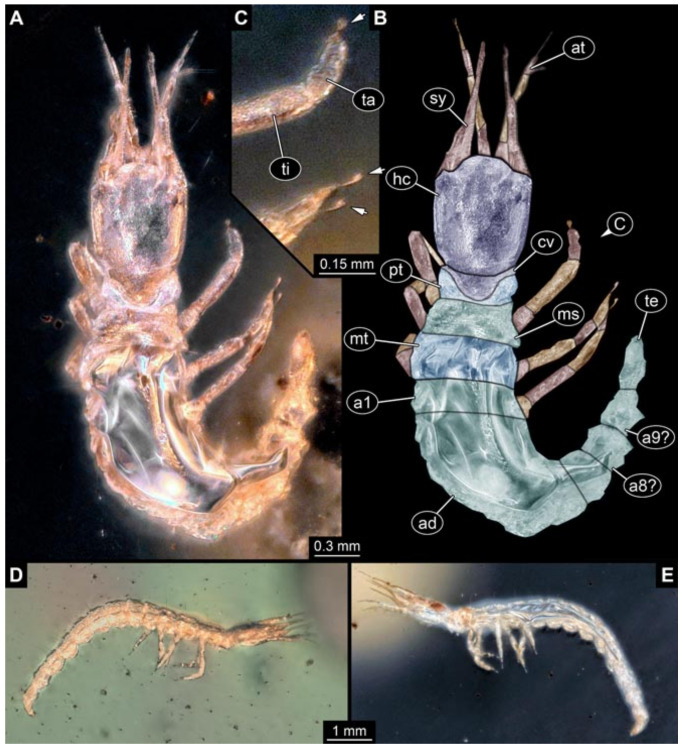
Specimens in Myanmar amber (**A**–**C**) Specimen 5823 (BUB 3064-1). (**A**) Dorsal view. (**B**) Dorsal view, colour marked. (**C**) Close-up of trunk appendage with empodium (arrows). (**D**–**E**) Specimen 5824 (BUB 3064-2). (**D**) Lateral view, slightly dorsally. (**E**) Lateral view, slightly ventrally. Abbreviations: a1 = abdomen segment 1; a8?–a9? = possible abdomen segments 8–9; ad = abdomen; at = antenna; cv = cervix; hc = head capsule; ms = mesothorax; mt = metathorax; pt = prothorax; sy = stylet; ta = tarsus; te = trunk end; ti = tibia.

**Figure 5:**
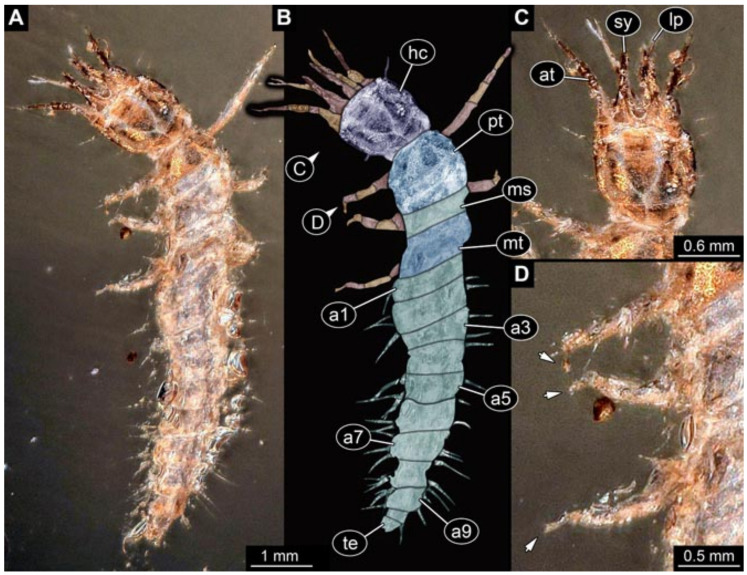
Specimen 5825 (BUB 3065); Myanmar amber. (**A**) Dorsal view. (**B**) Dorsal view, colour-marked. (**C**) Close-up of head capsule in dorsal view. (**D**) Close-up of trunk appendages with empodia (arrows). Abbreviations: a1–a9 = abdomen segments 1–9; at = antenna; hc = head capsule; lp = labial palp; ms = mesothorax; mt = metathorax; pt = prothorax; sy = stylet; te = trunk end.

**Figure 6:**
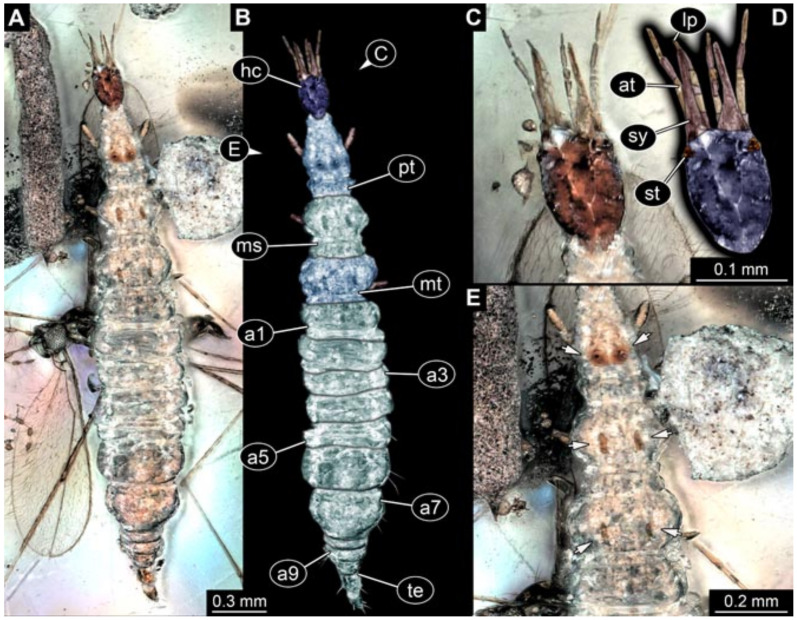
Specimen 5826 (BUB 3144); Myanmar amber. (**A**) Dorsal view. (**B**) Dorsal view, colour-marked. (**C**) Close-up of head capsule in dorsal view. (**D**) Close-up of head capsule in dorsal view, colour-marked. (**E**) Close-up of anterior trunk region with dark patches (arrows) in dorsal view. Abbreviations: a1–a9 = abdomen segments 1–9; at = antenna; hc = head capsule; lp = labial palp; ms = mesothorax; mt = metathorax; pt = prothorax; st = stemmata; sy = stylet; te = trunk end.

**Figure 7:**
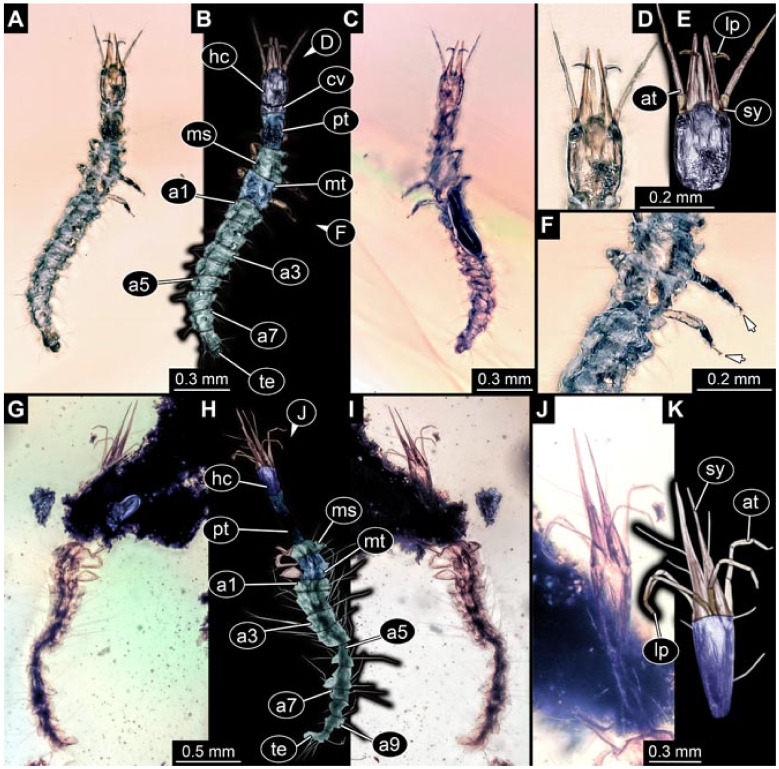
Specimens in Myanmar amber. (**A**–**F**) Specimen 5827 (BUB 3355). (**A**) Dorsal view. (**B**) Dorsal view, colour-marked. (**C**) Ventral view. (**D**) Close-up of head capsule in dorsal view. (**E**) Close-up of head capsule in dorsal view, colour-marked. (**F**) Close-up of trunk appendages with empodia (arrows). (**G**–**K**) Specimen 5828 (BUB 3390). (**G**) Supposed dorsal view. (**H**) Supposed ventral view, colour-marked. (**I**) Supposed ventral view. (**J**) Close-up of head in ventral view. (**K**) Close-up of head in ventral view, colour-marked. Abbreviations: a1–a9 = abdomen segment 1–9; at = antenna; cv = cervix; hc = head capsule; lp = labial palp; ms = mesothorax; mt = metathorax; pt = prothorax; sy = stylet; te = trunk end.

**Figure 8:**
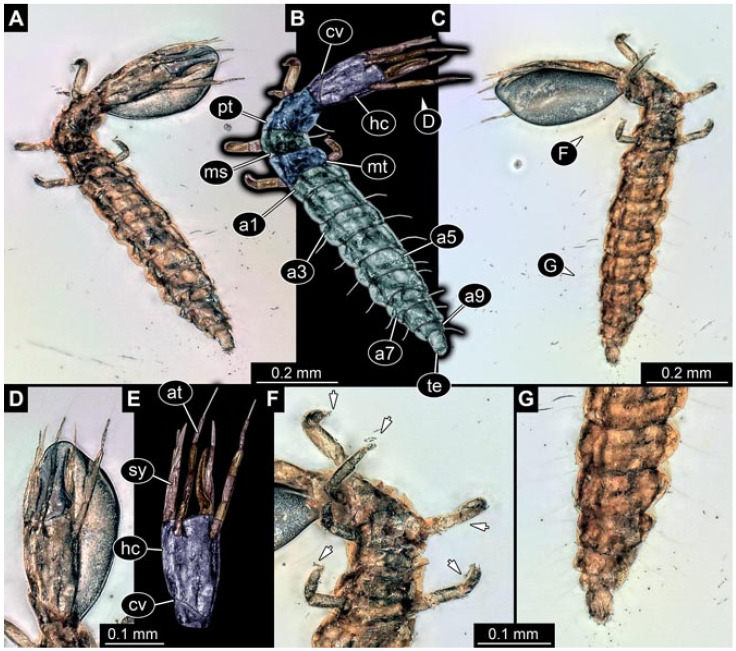
Specimen 5829 (BUB 3726); Myanmar amber. (**A**) Dorsal view. (**B**) Dorsal view, colour-marked. (**C**) Ventral view. (**D**) Close-up of head in dorsal view. (**E**) Close-up of head in dorsal view, colour-marked. (**F**) Close-up of trunk appendages with empodia (arrows). (**G**) Close-up of trunk end in ventral view. Abbreviations: a1–a9 = abdomen segments 1–9; at = antenna; cv = cervix; hc = head capsule; ms = mesothorax; mt = metathorax; pt = prothorax; sy = stylet; te = trunk end.

**Figure 9:**
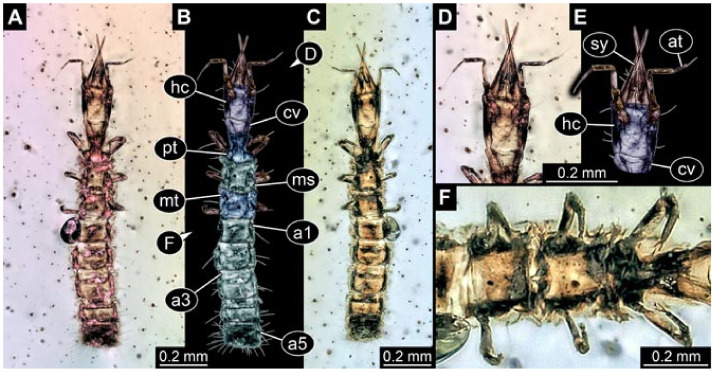
Specimen 5830 (BUB 3741); Myanmar amber. (**A**) Dorsal view. (**B**) Dorsal view, colour-marked. (**C**) Ventral view. (**D**) Close-up of head in dorsal view. (**E**) Close-up of head in dorsal view, colour-marked. (**F**) Close-up of trunk appendages. Abbreviations: a1–a5 = abdomen segments 1–5; at = antenna; cv = cervix; hc = head capsule; ms = mesothorax; mt = metathorax; pt = prothorax; sy = stylet.

**Figure 10:**
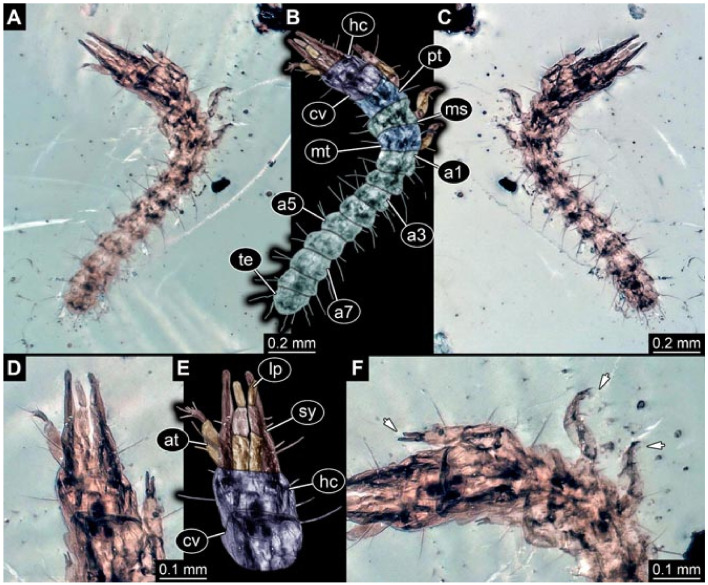
Specimen 5831 (BUB 3962); Myanmar amber. (**A**) Dorsal view. (**B**) Dorsal view, colour-marked. (**C**) Ventral view. (**D**) Close-up of head in dorsal view. (**E**) Close-up of head in dorsal view, colour-marked. (**F**) Close-up of trunk appendages with empodia (arrows) in ventral view. Abbreviations: a1–a7 = abdomen segments 1–7; at = antenna; cv = cervix; hc = head capsule; lp = labial palp; ms = mesothorax; mt = metathorax; pt = prothorax; sy = stylet; te = trunk end.

**Figure 11:**
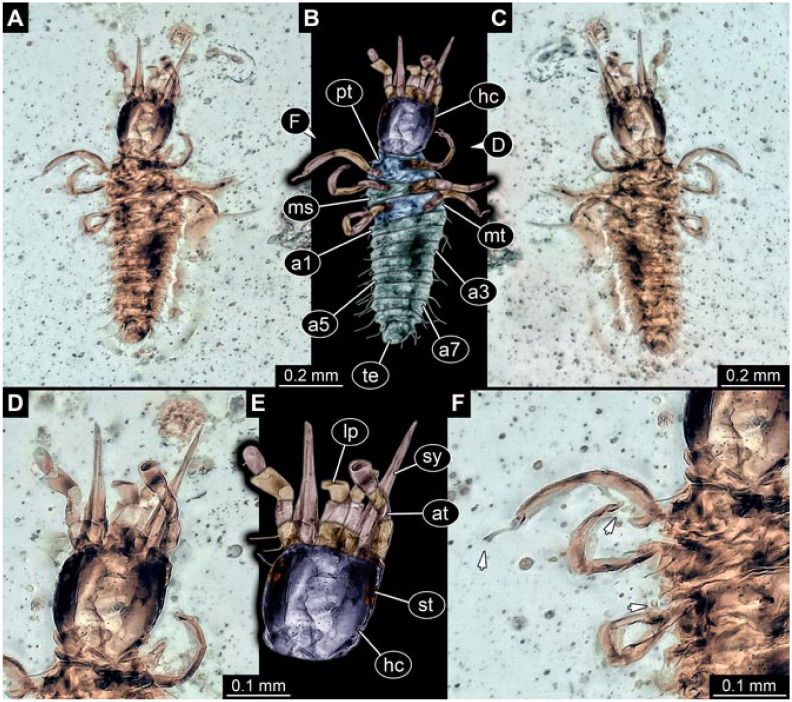
Specimen 5832 (BUB 3963); Myanmar amber. (**A**) Ventral view. (**B**) Ventral view, colour-marked. (**C**) Dorsal view. (**D**) Close-up of head in ventral view. (**E**) Close-up of head in ventral view, colour-marked. (**F**) Close-up of trunk appendages with empodia (arrows) in ventral view. Abbreviations: a1–a7 = abdomen segments 1–7; at = antenna; hc = head capsule; lp = labial palp; ms = mesothorax; mt = metathorax; pt = prothorax; st = stemmata; sy = stylet; te = trunk end.

**Figure 12:**
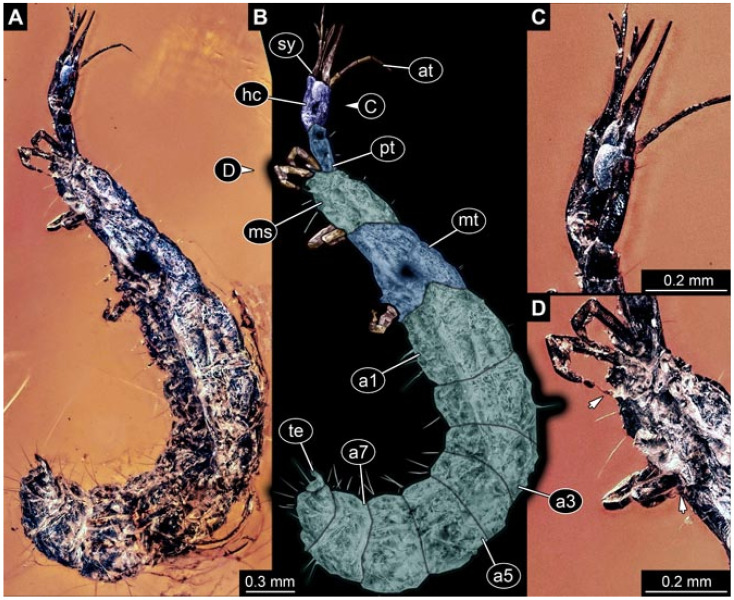
Specimen 5833 (CJW F 3197); Myanmar amber. (**A**) Lateral view slightly curved. (**B**) Lateral view slightly curved, colour-marked. (**C**) Close-up of head in dorso-lateral view. (**D**) Close-up of trunk appendages with empodia (arrows) in ventro-lateral view. Abbreviations: a1–a7 = abdomen segments 1–7; at = antenna; hc = head capsule; ms = mesothorax; mt = metathorax; pt = prothorax; sy = stylet; te = trunk end.

**Figure 13:**
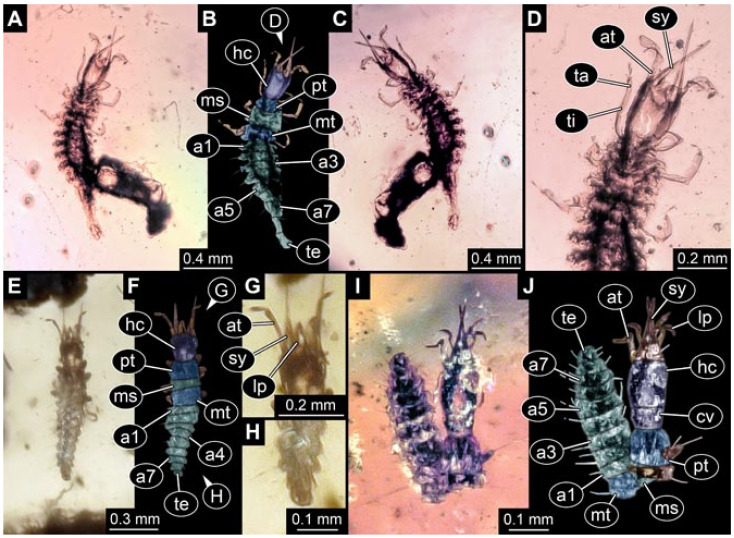
Specimens in Myanmar amber. (**A**–**D**) Specimen 5834 (PED 0380). (**A**) Dorsal view. (**B**) Dorsal view, colour-marked. (**C**) Ventral view. (**D**) Close-up of head and trunk appendages in dorsal view. (**E**–**H**) Specimen 5835 (CJW F 3198). (**E**) Dorsal view. (**F**) Dorsal view, colour-marked. (**G**) Close-up of head in dorsal view. (**H**) Close-up of trunk end in dorsal view. (**I**–**J**) Specimen 5836 (CJW F 3336). (**I**) Dorsal view. (**J**) Dorsal view, colour-marked. Abbreviations: a1–a7 = abdomen segment 1–7; at = antenna; cv = cervix; hc = head capsule; lp = labial palp; ms = mesothorax; mt = metathorax; pt = prothorax; sy = stylet; ta = tarsus; te = trunk end; ti = tibia.

**Figure 14:**
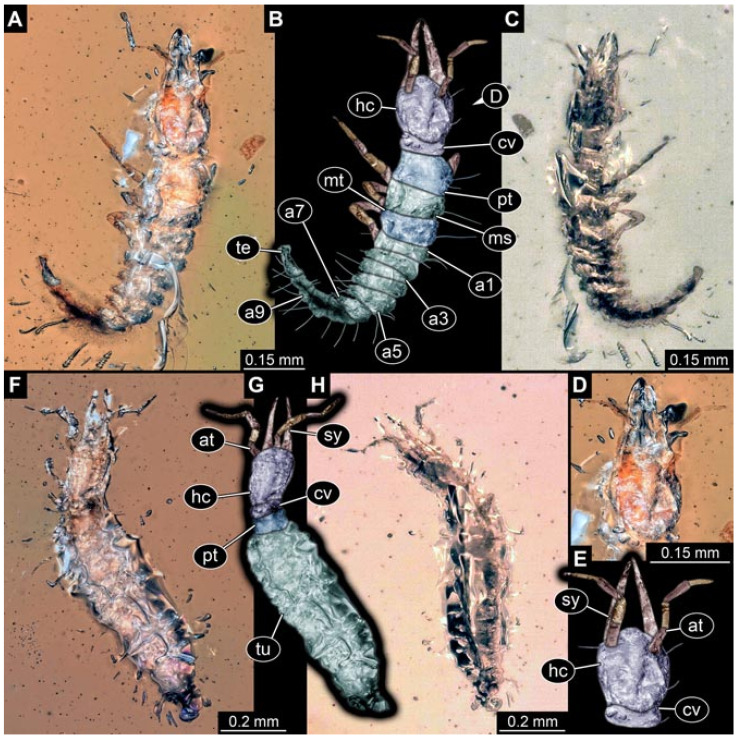
Specimens in Myanmar amber. (**A**–**E**) Specimen 5837 (PED 0772-1). (**A**) Dorsal view. (**B**) Dorsal view, colour-marked. (**C**) Ventral view. (**D**) Close-up of head in dorsal view. (**E**) Close-up of head in dorsal view, colour-marked. (**F**–**H**) Specimen 5838 (PED 0772-2). (**F**) Dorsal view. (**G**) Dorsal view, colour-marked. (**H**) Ventral view. Abbreviations: a1–a9 = abdomen segment 1–9; at = antenna; cv = cervix; hc = head capsule; ms = mesothorax; mt = metathorax; pt = prothorax; sy = stylet; te = trunk end; tu = trunk.

**Figure 15:**
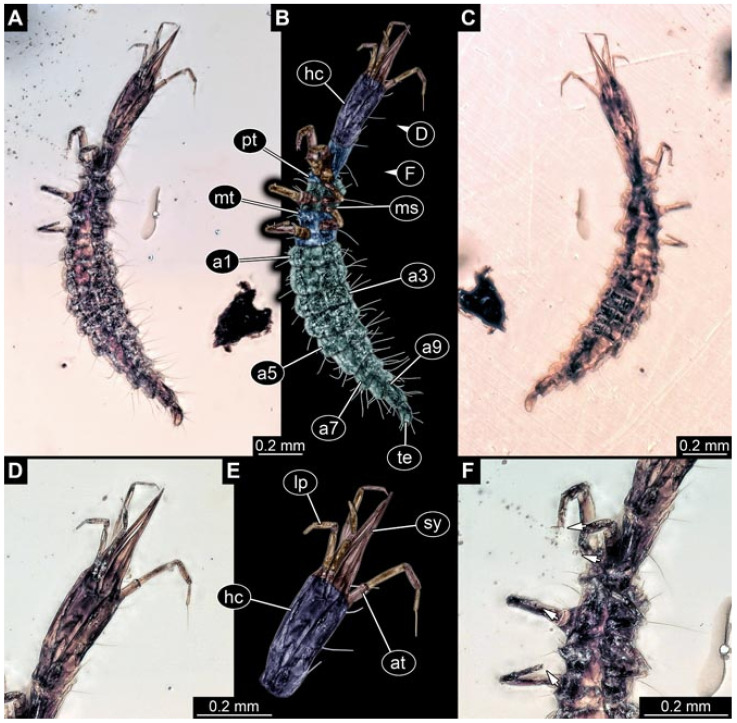
Specimen 5839 (PED 0828); Myanmar amber. (**A**) Ventral view. (**B**) Ventral view, colour-marked. (**C**) Dorsal view. (**D**) Close-up of head in ventral view. (**E**) Close-up of head in ventral view, colour-marked. (**F**) Close-up of trunk appendages with empodia (arrows) in ventral view. Abbreviations: a1–a9 = abdomen segments 1–9; at = antenna; hc = head capsule; lp = labial palp; ms = mesothorax; mt = metathorax; pt = prothorax; sy = stylet; te = trunk end.

(1) Brauer [53] depicted a drawing of a stage 1 larva (hatchling) of *Mantispa pagana* (specimen 5201) in dorsal view (his Figure 1 pl. 1). No indication of size was provided. Parts of the specimen seem to have been re-figured in Stitz [54] and Jacobs [55]. The specimen was re-figured in Aspöck [56].
(2) Brauer [57] provided drawings of a stage 1 larva (hatchling) of *Mantispa pagana* (specimen 5202). The images include the hatching process (his Figure 11 pl. IV), habitus in lateral (his Figure 11e pl. IV), and dorsal view (his Figure 11’’ pl. IV), trunk appendages (his Figure 11a,b pl. IV), and the head in dorsal view (his Figure 11c pl. IV). Parts of the specimen seem to have been re-figured in Stitz [54] and Jacobs [55]. The entire plate with all figures was re-figured in Aspöck [56].
(3) Brauer [58] provided several drawings of larvae of *Mantispa styriaca*. Images include a stage 1 larva (hatchling) in dorsal view (his Figure 1 pl. XII), stage 1 larvae entering the egg sac of a spider (his Figure 2 pl. XII), and later stage larvae including some details of the head (his Figures 3–4 pl. XII). Yet, only the stage 1 larva in dorsal view has sufficient details to be further considered, which seems quite similar to specimen 5202, i.e., the one from Brauer [57]. Yet, a closer comparison shows significant differences indicating that this is another specimen (specimen 5203).
  Parts of this specimen seem to have been re-figured in Stitz [54] and Jacobs [55]. Some of the later stage larvae were re-figured by Poivre [59]. One of the laterstage larvae has been re-figured in Aspöck and Aspöck [60], Gepp [24], and Makarkin [61]. The entire plate with all figures was re-figured in Aspöck [56] and Aspöck and Aspöck [3].
(4) Nawa [62] provided a very simplified drawing of a larva of Mantispidae. The text is in Japanese and could not be translated with common tools. Based on later publications [23], the specimen was a stage 1 larva of *Eumantispa harmondi*. The drawing appears very simplified (MacLeod [23] referred to it as “crude”, p. 263). The stylet-head-capsule transition is not shown, not permitting the standardization of the orientation of the stylets. Therefore, the specimen could not be further considered.
(5) Tillyard [37] provided a drawing of a stage 1 larva (hatchling) of *Mantispa vittata* (specimen 5204) in dorsal view (his Figure U13A p. 319). The size of the larva was provided as a magnification factor, which is not informative in combination with the electronic versions available to the present authors. Tillyard [37] also provided a drawing of a stage 3 larva (his Figure U13B p. 319), but in lateral view, hence this specimen could not be further considered here. Both specimens were re-figured by Tjeder [39].
(6) Stitz [54] provided a drawing (his Figure 331 p. 35.302) of a larva of *Mantispa pagana.* In the figure legend it was stated that the figure is redrawn from “Brauer”. In the reference list, Stitz [54] cites three possible sources: Brauer [53] (specimen 5201), Brauer [57] (specimen 5202), and Brauer [58] (specimen 5203). A close comparison shows no direct match to any of the three possible specimens. Most likely, the drawing by Stitz [54] combined information from two of these or even all three specimens. The drawing is therefore still interpreted as a re-figure and was not further considered.
  Stitz [54] additionally figured a stage 2 larva in lateral view (his Figure 332 p. 35.302) and a close-up of the head in lateral view (his Figure 333 p. 35.302). Due to the orientation, this specimen could not be further considered.
(7) Handschin [63] provided drawings of a stage 1 larva (hatchling) of *Austromantispa imbecilla* (specimen 5205). Images include a head in dorsal view (his Figure 17 left p. 706) and the isolated labium (his Figure 17 right p. 706). No indication of size was provided.
(8) Hungerford [64] provided a micrograph of a stage 1 larva (specimen 5206) of *Mantispa interrupta* in dorsal view (his Figure 4 pl. 1). The size of the specimen was provided as a magnification factor, which is not informative in combination with the electronic versions available to the present authors. According to a re-figured version [24] (see below), the larva was about 1 mm long.
(9) Merti [65] provided a drawing of a stage 1 larva (specimen 5207) of *Mantispa decorata* in dorsal view (his Figure 3 p. 306). No indication of size was provided.
(10) McKeown and Mincham [66] provided a drawing of a stage 1 larva of *Mantispa vittata* (specimen 5208) in dorsal view (their Figure 8 p. 217). No direct indication of size was provided. According to the text, the larvae that the drawing is based on were slightly larger than 1 mm (p. 212). Images of later-stage larvae were provided (their Figures 9 and 10 p. 217), unfortunately only in lateral view or without sufficient details; hence, these could not be further considered.
(11) Peterson [22] provided a drawing of a stage 1 larva of *Mantispa interrupta* (specimen 5209) in dorsal view (his Figure N2A). Apparently, there are later editions and reprints of this work. These have not all been viewed by the current authors (the edition available was labelled 1957), but we can assume that they also contain the image. Hence, citations of these later editions are likely to refer to this specimen. The exact year of publication of the different editions or reprints, is unclear. We found reference to the years 1953, 1956, 1957, 1960, 1962, 1965, 1967, and 1979.
(12) Lucchese [67] provided several drawings of stage 1 larva (specimen 5210) of *Mantispa perla*. Drawings include a dorsal view (his Figure 11 left p. 254), a lateral view (his Figure 11 right p. 254), and a number of details of the trunk appendages (his Figure 12 p. 255). No indication of size was provided.
(13) Lucchese [68] re-figured (his Figures XXXIII p. 134, XL p. 142) specimen 5210, i.e., all images from Lucchese [67]. Additional views and details of this specimen were provided as drawings, including a ventral view (his Figure XXXIV p. 131), the head in dorsal (his Figure XXXV p. 132), ventral (his Figure XXXVI p. 135), and lateral view (his Figure XXXVII p. 137), antenna and labial palp (his Figure XXXVIII p. 139), details of the stylet (his Figure XXXIX p. 140), and the trunk end in dorsal and ventral view (his Figure XLI p. 143). In addition, the entire hatching process was presented in lateral view (Figure LII p. 173, LIII p. 175, LIV p. 177, LV p. 179). The ventral view was re-figured in Tauber [40].
(14) Tjeder [39] re-figured specimen 5204 (his Figure 219 p. 259) and another larva in lateral view (his Figure 220), i.e., the two drawings from Tillyard [37].
(15) Kuroko [69] provided several images of larvae of Mantispidae. The first one (specimen 5211) was a stage 1 larva of *Climaciella magna*. The images include drawings of the entire larva in dorsal view (his Figure 3 pl. 10), the head in dorsal view (his Figure 4 pl. 10), trunk appendage (his Figure 5 pl. 10), head in ventral view (his Figure 6 pl. 10), mandibles (his Figure 7 pl. 10), trunk end (his Figure 8 pl. 10), and a scheme of the setae arrangement (chaetotaxy; his Figure 9 pl. 10). According to the provided scale, the larva was 1 mm long.
  The second specimen (specimen 5212) was a stage 1 larva of Mantispa japonica. The images include drawings of the entire larva in dorsal view (his Figure 10 pl. 11), a scheme of the setae arrangement (chaetotaxy; his Figure 11 pl. 11), maxillae and labium (his Figure 12 pl. 11), a dorsal view of the head (his Figure 13 pl. 11), mandibles (his Figure 14 pl. 11), and a trunk appendage (his Figure 15 pl. 11). According to the provided scale, the larva was 1 mm long.
  Kuroko [69] also provided a micrograph of many stage 1 larvae of *Climaciella magna*. Yet, the image does not provide sufficient details. Hence, it could not be further considered.
(16) Ghilarov [70] provided a drawing (his Figure 12 p. 411) of a stage 1 larva (specimen 5213) of Mantispa styriaca in dorsal view. No indication of size was provided. Additionally, a stage 3 larva was shown in lateral view (his Figure 39.3 p. 49), but could not be further considered due to its orientation. Both images were re-figured by Dorohova [71].
(17) Aspöck and Aspöck [60] re-figured (their Figure 98 p. 279) a lateral view of a stage 2 larva, i.e., a drawing from Brauer [58], but citing Brauer [53].
(18) MacLeod [23] provided drawings of several larvae of Mantispidae. The first drawing (specimen 5214) was of the head of a stage 1 larva of Plega melitomae in dorsal (his Figure 42 pl. XIV) and ventral view (his Figure 44 pl XIV). Additionally, a detail of the antenna was provided (his Figure 43 pl. XIV). This larva differs from other larvae by possessing slightly curved stylets. According to the provided scale, the head capsule was 0.21 mm long. The specimen was re-figured in Haug et al. [12].
  The second one (specimen 5215) was a drawing of the head of a stage 1 larva of Mantispa viridis in dorsal (his Figure 49 pl. XVI) and ventral view (his Figure 51pl XVI). Additionally, details of the head capsule surface (his Figure 48 pl. XVI), antenna (his Figure 50 pl. XVI), mandible (his Figure 52 pl. XVI), and head in lateral view (his Figure 53 pl. XVI) were provided. According to the provided scale, the head capsule was 0.13 mm long. The specimen was re-figured in Engel et al. [72].
  The third drawing (specimen 5216) was of a head of a stage 3 larva of an unidentified representative of Mantispidae in dorsal (his Figure 54 pl. XVII) and ventral view (his Figure 55 pl. XVII). According to the provided scale, the head capsule was 0.31 mm long.
(19) Parker and Stange [73] provided drawings of two larvae of Plega yucatanae. This first drawing was of a head of a stage 1 larva (specimen 5217) in dorsal view (their Figure 10 p. 610). No indication of size was provided.
  The second drawing was of a stage 3 larva (specimen 5218). Images include a head in dorsal view (their Figure 11 p. 610) and the habitus in lateral view (their Figure 12 p. 610). The entire larva was, according to the text, 10 mm long. The head of the specimen was re-figured in Tauber [40].
(20) Bissett and Moran [74] provided a drawing of a stage 1 larva (hatchling) of an unidentified representative of Mantispidae (specimen 5219) in dorsal view (their Figure 2a p. 85). Additionally, details of the stylets (their Figure 2b), antenna (their Figure 2c), labium (their Figure 2d), and trunk appendages (their Figure 2e) were provided. According to the provided scale, the specimen was 0.95 mm long.
(21) Davidson [75] provided drawings of several larvae of *Mantispa viridis* (all on p. 31). Two of the drawings show entire larvae in dorsal view, and two drawings show isolated heads in dorsal view. Two drawings (one of a head and one habitus drawing) were labelled as being based on a stage 1 larva (his Figures 1 and 3). We interpret these as representing the same specimen (specimen 5220). The other drawing of a head was labelled as a stage 2 larva (his Figure 2), and the other habitus drawing was labelled as a stage 4 larva (his Figure 4). As there is no stage 4 larva in Mantispidae (also not mentioned in the text), we assume that this was a mistake. We therefore interpret these two drawings as representing a single specimen (specimen 5221).
(22) Riek [1] provided drawings of a larva of *Mantispa* sp. The images include the habitus in lateral view (his Figure 29.11A p. 489) and a dorsal view of the head region (his Figure 29.11B p. 489), which therefore could be considered for the analysis here (specimen 5222). The larva was labelled as a stage 1 larva; yet, the morphology is more likely that of a stage 2 or 3 larva, especially based on the head detail. No indication of size was provided. The specimen was re-figured in Gepp [24], New [43], Tauber et al. [44], and Heckman [51].
(23) Anonymus [76] provided drawings of stage 1, 2, and 3 larvae of Mantispidae. The stage 1 larva was shown in dorsal view. Although the drawing appears rather simplified, it differs from all earlier images reported above and provides sufficient detail to be further considered (specimen 5223). The stage 2 and 3 larvae were shown in lateral view and could therefore not be further considered.
(24) Poivre [59] provided a drawing of a stage 1 larva (specimen 5224) of *Mantispa styriaca* in dorsal view (his Figure 2A p. 7) and during the hatching process (his Figure 2C–E p. 7). According to the provided scale, the specimen was 1 mm long. The specimen was re-figured by Makarkin [61]. Poivre [59] additionally re-figured (his Figure 2K,L p. 7) some later-stage larvae from Brauer [58].
(25) Redborg [77] depicted a larva of *Mantispa uhleri* parasitising a spider (his Figure 1A p. 190). Yet, the larva is not very well visible and could not be considered further here.
(26) Gepp [24] re-figured three specimens: a later-stage larva (his Figure 9a pl. 4 p. 195), i.e., a specimen from Brauer [58]; specimen 5206 (his Figure 9b pl. 4 p. 195), i.e., the specimen from Hungerford [64]; and specimen 5222 (Figure 9c pl. 4 p. 195), i.e., the specimen from Riek [1].
(27) Redborg and MacLeod [78] provided numerous images of larvae of *Mantispa uhleri*. The images included drawings of a stage 1 larva (hatchling) in dorsal view (their Figure 4A p. 12; specimen 5225), the head of a stage 3 larva in lateral view (their Figure 4B p. 12; not further considered), the trunk end of a stage 2 larva in lateral view (their Figure 4C p. 12; not further considered), the trunk end of a stage 3 larva in lateral view (their Figure 4D p. 12; not further considered), a stage 3 larva in lateral view (their Figure 6 p. 17; not further considered), as well as micrographs of stage 1 larvae on spiders (their Figure 13 p. 65; their Figure 14 p. 67; not further considered). According to the provided scale, the considered single stage 1 larva specimen was 0.88 mm long. Some images were re-figured in Tauber [40] and Haug et al. [12].
(28) Tauber [40] re-figured several specimens. The first one (her Figure 33.14 p. 134) was specimen 5225, i.e., a specimen from Redborg and MacLeod [78], but Redborg [77] was cited as the source. The second one was the ventral view of the head of specimen 5210 (her Figure 33.15 p. 134), i.e., a drawing from Lucchese [68], but citing Luchcese [67] as the source. The third one was a stage 3 larva in lateral view (her Figure 33.16 p. 134), i.e., a drawing from Redborg and MacLeod [78], but Redborg [77] was cited as the source. The fourth one (her Figure 33.17 p. 134) was the head of specimen 5218, i.e., a drawing from Parker and Stange [73]. Apparently, there are later editions of this contribution also figuring this image (at least 1987, 1991, 2008, our version was labelled 1991). We assume that all of these feature the same images. Hence, these also refer to the same specimen.
(29) Dorohova [71] re-figured the drawings from Ghilarov [70], i.e., specimen 5213 (her Figure 39.2 p. 49) and the stage 3 larva in lateral view (her Figure 39.3 p. 49).
(30) Monserrat and Díaz-Aranda [79] provided images of several larvae of *Mantispa styriaca*. These include a stage 1 larva in lateral view (their Figure 1 p. 194) and numerous details (their Figures 2 and 3 p. 194, their Figures 4–7 p. 195, their Figures 8–11 p. 196). Yet, no dorsal depiction of the head with stylets is available, and hence this specimen could not be further considered here.
  The second specimen was a stage 3 larva shown in lateral view (their Figure 12 p. 197), also with details (their Figures 14 and 15 p. 197), and a head with stylets in dorsal view (their Figure 13 p. 197). Therefore, this specimen (specimen 5226) could be considered in the analysis here.
(31) New [25] re-figured specimen 5222 (his Figure 141 p. 100), i.e., the specimen from Riek [1].
(32) Kral [80] provided a partial SEM image of a stage 1 larva of *Mantispa*. The image shows the head in more or less dorsal view, yet it is too incomplete to be further considered here.
(33) Minter [46] provided drawings of a stage 1 larva (specimen 5227) of *Mantispa capeneri*. The images include the habitus in dorsal view (his Figure 23 p. 125) and the head in dorsal (his Figure 24 p. 125) and ventral view (his Figure 25 p. 125). Additionally, SEM micrographs of several details were provided including eyes (his Figure 26 p. 126), empodium (his Figure 27 p. 126), surface of head capsule (his Figure 28 p. 126), and trunk (his Figure 29 p. 16). According to the provided scale, the larva was 0.76 mm long. The specimen was re-figured in Aspöck and Aspöck [3].
(34) New [43] apparently re-figured specimen 5222, i.e., the specimen from Riek [1]. This source was not directly seen by the current authors and the information is based on a reference in Tauber et al. [44] and Heckman [51]. Therefore, the figure number remains unknown.
(35) Hoffman and Brushwein [81] provided numerous drawings of larvae of Mantispidae. These included the habitus of an entire stage 1 larva of Mantispa pulchella (their Figure 1 p. 183; partly simplified), and a simplified ventral view (their Figure 2 p. 183); simplified dorsal views of stage 1 larvae of Mantispa interrupta (their Figure 3 p. 183), Mantispa sayi (their Figure 4 p. 183), and Mantispa viridis (their Figure 5 p. 183); all these are rather simplified in the head region and can therefore not be used for the analysis performed herein. More detailed images were also provided.
  The first one was a detailed drawing of a head of a stage 1 larva (specimen 5228) of *Climaciella brunnea* in dorsal and ventral view (their Figure 6 p. 184), as well as details of the trunk appendages (their Figure 7 p. 184) and setae arrangement (their Figure 8 p. 185).
  The second one was a detailed drawing of a head of a stage 1 larva (specimen 5229) of Mantispa interrupta in dorsal and ventral view (their Figure 9 p. 186), as well as details of the trunk appendages (their Figure 10 p. 186) and setae arrangement (their Figure 11 p. 187). In addition, there were different details (e.g., trunk end) of stage 2 and 3 larvae (their Figures 12–14 p. 188) that could not be further considered here.
  The third one is a detailed drawing of a head of a stage 1 larva (specimen 5230) of *Mantispa pulchella* in dorsal and ventral view (their Figure 15 p. 189), as well as details of the trunk appendages (their Figure 16 p. 189) and setae arrangement (their Figure 17 p. 190). In addition, there were details of stage 2 and 3 larvae (their Figure 18 p. 191, their Figure 19 p. 192) that could not be further considered here due to the orientation of these images. Some of the images were re-figured in Ohl [11]. Parts of the head were re-figured in Haug et al. [12].
  The fourth one was a detailed drawing of a head of a stage 1 larva (specimen 5231) of Mantispa viridis in dorsal and ventral view (their Figure 21 p. 194), as well as details of the trunk appendages (their Figure 22 p. 194) and setae arrangement (their Figure 23 p. 195). In addition, there were details (e.g., trunk end) of stage 2 and 3 larvae (their Figures 24–26 p. 196) that could not be further considered here, as no head was shown in dorsal view.
  For all of the specimens that could be considered here, no indication of size was provided.
(36) Hirata et al. [82] provided micrographs of stage 1 larvae of *Eumantispa harmandi* on a spider (their Figure 1). Yet, none of the specimens were available in dorsal view, hence these specimens could not be further considered here.
(37) Makarkin [61] re-figured specimen 5224 (his Figure 17.5 p. 43), i.e., the drawing from Poivre [59]. He also re-figured a stage 3 larva from Brauer [58].
(38) Jacobs [55] re-figured a stage 2 larva in lateral view (his Figure M-10 right p. 364), i.e., a larva from Aspöck and Aspöck [60]. In addition, Jacobs [55] figured a stage 1 larva of *Mantispa styriaca* (his Figure M-10 left p. 364), which is a re-figure of the compound drawing from Stitz [54], without reference.
(39) Aspöck [56] re-figured several specimens from historically important studies. The images include specimen 5201 (his Figure 61 p. 233), i.e., the specimen from Brauer [53]; specimen 5202 (his Figure 67 p. 235), i.e., the specimen (entire plate) from Brauer [57]; and specimen 5203 (his Figure 71 p. 237), i.e., the specimen (entire plate) from Brauer [58].
(40) Tauber et al. [44] re-figured specimen 5222 (their Figure 6J p. 793), i.e., the specimen from Riek [1], but citing New [43] as the source.
(41) Aspöck and Aspöck [3] provided a photograph of a stage 1 larva of *Mantispa scabricollis*. Unfortunately, the image does not provide sufficient details to be further considered here.
  Aspöck and Aspöck [3] also re-figured (their Figure 94, p. 487) a ventral view of the head of specimen 5227, i.e., the specimen from Minter [46]; and specimen 5203 (their Figure 108 p. 491), i.e., the drawing from Brauer [58], together with all other figures on the same plate.
(42) Ohl [11] re-figured two drawings from Hoffman and Brushwein [81], a stage 1 larva of *Leptomantispa* pulchella (his Figure 2b p. 455) and a stage 3 larva of the same species (his Figure 2b p. 455).
(43) Wunderlich [83] re-figured (his Figure 3 p. 156) a specimen from Kuroko [69]. The size was stated as 10 mm (“1 cm”), which was likely a calculation error as the original size was indicated to be 1 mm. Wunderlich also re-figured a stage 3 larva of *Leptomantispa pulchella* (his Figure 4 p. 156), i.e., a larva from Hoffman and Brushwein [81], but cited it as “*Mantispa* sp.” with Ohl [11] as source.
(44) Monserrat [84] re-figured the two specimens from Monserrat and Diaz-Aranda [79].
(45) Nagler and Haug [85] re-figured a specimen (their Figure 3e p. 155) from Hoffman and Brushwein [81], but cited from a secondary source [11].
(46) Kral and Devetak [86] re-figured (their Figure 1 p. 246) the images of Kral [80]. The images are incomplete and could not be further considered.
(47) Dorey and Merritt [87] provided micrographs of stage 1 larvae of *Ditaxis biseriata*. These include images of a group of newly hatched specimens (their Figure 3 upper right p. 6), a specimen preserved in ethanol in dorsal view (their Figure 3 middle p. 6), and a living specimen in dorsal view (their Figure 3 lower p. 6). None of these images provided sufficient details to be further considered here. More detailed images include micrographs of an antenna (their Figure 4a p. 7) and a head illuminated with transmitted light (their Figure 4b p. 7), as well as a head in dorsal view illuminated with reflected light (their Figure 4c p. 7). It remains unclear whether the two depictions of the head are from the same specimen. As we cannot exclude that it is the same specimen, we consider them as one specimen (specimen 5232) in order to avoid considering the same specimen twice. According to the provided scale, the head capsule of the specimen was 0.25 mm long.
(48) Heckman [51] re-figured specimen 5222 (his Figure 2.36), the specimen from Riek [1], but cited New [43] as the source.
(49) Haug et al. [12] re-figured several larvae of Mantispidae. The images include specimen 5225 (their Figure 3B p. 5), i.e., a specimen from Redborg and MacLeod [78]; details of the antenna and labial palp of specimen 5230 (their Figure 3C,D p. 5), i.e., a specimen from Hoffman and Brushwein [81]; and the head of specimen 5214 (their Figure 3E p. 5), i.e., a specimen from MacLeod [23].
(50) Engel et al. [72] re-figured specimen 5215 (their Figure 3g p. 541), i.e., a drawing from MacLeod [23].
(51) Jandausch et al. [88] provided an intensive documentation of stage 1 larvae of *Mantispa aphavexelte*. These images included:
  (1) SEM micrographs of an entire specimen in dorsal (their Figure 1A p. 532), ventral (their Figure 1B p. 532), and lateral view (their Figure 1C p. 532), close-ups of the head in dorsal (their Figure 3A p. 533), ventral (their Figure 3B p. 533), lateral (their Figure 3C p. 533), and anterior view (their Figure 3D p. 533), eyes (their Figure 4 p. 534), antennae (their Figure 5 p. 534), mouthparts in general (their Figure 6A p. 535), details of the stylets (their Figure 6B–D p. 535), the labial palp (their Figure 8 p. 536), and the thorax in lateral view (their Figure 13A p. 542).
  (2) Light microscopic photographs of an entire specimen in dorsal (their Figure 2A p. 532), ventral (their Figure 2B p. 532), and lateral view (their Figure 2C p. 532), and histological sections through different body regions (their Figure 9 p. 539).
  (3) Surface renderings of 3D models of the stylets (their Figure 7 p. 536), the head (their Figure 10 p. 539), the nervous system (their Figure 11A p. 540), and digestive tract (their Figure 11B p. 540), the musculature of the foregut (their Figure 12 p. 540), and the trunk muscles (their Figure 14 p. 543, their Figure 16 p. 545, their Figure 17 p. 546).
  (4) Drawings of a larva in lateral view (their Figure 9 p. 538 upper), the thorax (their Figure 13B p. 542), and the anterior abdomen (their Figure 15 p. 544), both in lateral view, and the head in dorsal (their Figure 18A p. 548) and ventral view (their Figure 18B p. 548).
    It remains unclear which individual was used as the basis for each image. We therefore considered only a single specimen (their Figure 18A; specimen 5233) in order to avoid considering the same specimen twice. According to the provided scale, the head capsule of the specimen was 120 µm long.
    Jandausch et al. [88] additionally re-figured a drawing of a stage 3 larva of *Dicromantispa sayi*. According to the figure legend, the original drawing is from “Redborg (1982)”, but the reference was not found in the reference list. We found two publications by Redborg from 1982 in the literature, none of which feature a comparable drawing [77,89]. As another source of the same drawing, “Stehr (1987)”, was cited, but also not found in the reference list. Comparison to known images revealed a high match with a drawing from Redborg and MacLeod [78].
(52) Jandausch et al. [90] re-figured images from Jandausch et al. [88], providing additional details. The re-figured image was an SEM micrograph showing the habitus in lateral view (their Figure 1) of the stage 1 larva of Mantispa aphavexelte. Additional details include aspects of the trunk appendages, including drawings (their Figures 2 and 4), SEM micrographs (their Figure 3), and cLSM-based micrographs (their Figure 5).
(53) Badano et al. [91] provided a photograph of a larva of Mantispidae in dorsal view (their Figure 4iii p. 679). Yet, is difficult to access details of the mouthparts and the posterior edge of the head capsule. Therefore, the specimen could not be further considered for our analysis.

### 3.3. Extant Larvae of Dilaridae 

In total, nine extant specimens of larvae of Dilaridae (specimens 5401–5409) were found in the literature that could be used for our analysis. These were presented in 20 publications, which are listed as follows.

(1) Takahashi [92] reported a larva and interpreted it as a larva of Dilaridae. Later studies demonstrated that this larva is one of the group Nevrorthidae, hence most likely of *Nipponeurorthus* (see recent discussion in [6]). Therefore, the specimen could not be further considered here.
(2) Gurney [38] provided several drawings of a stage 3 larva of *Nallachius americanus* (specimen 5401). The images include the habitus in dorsal view (his Figure 6 pl. 11 p. 163), the head in dorsal view (his Figure 11 pl. 12 p. 165), the head in ventral view (his Figure 14 pl. 13 p. 167), and details of the trunk appendages (his Figures 17 and 21 pl. 14 p. 169). The length of the entire larva was 12 mm. The specimen was re-figured in Peterson [22], Gepp [24], Tauber [40], and Tauber et al. [44].
(3) Peterson [22] provided several drawings of a larva of *Nalachius americanus*. Images include the habitus in dorsal view (his Figure N2B), the head in dorsal view (his Figure N2C), trunk appendages (his Figure N2D,E), and the terminal end (his Figure N2F). The figures refer to Gurney [38]. While the images differ from the original, they indeed seem to be based on Gurney [38], but modified. Hence, the images from Peterson [22] could not be further considered. There are later editions of this book (e.g., 1953, 1957, 1960, see also above) which seem to have used the same images; therefore, these also refer to the same specimen.
(4) Ghilarov [70] provided several drawings of a larva (specimen 5402) of *Dilar turcicus*. The images include the habitus in dorsal view (his Figure 1 p. 403), the head in ventral (his Figure 2a p. 404) and dorsal view, mandible (his Figure 3a p. 404), maxilla (his Figure 3b p. 404), antenna (his Figure 4 p. 404), labium (his Figure 5 p. 405), trunk appendage (his Figure 6a p. 405), tip of a trunk appendage (his Figure 6b p. 405), and the trunk end in dorsal and ventral view (his Figure 7 p. 405). No indication of size was provided. The specimen was re-figured by Gepp [24], Dorohova [71], and New [25].
(5) MacLeod [23] provided several drawings of the head of a stage 3 larva of *Nallachius americanus* (specimen 5403). The images include a lateral (his Figure 32 pl. X), a dorsal (his Figure 33 pl. XI), and a ventral view (his Figure 34 pl. XI), and an isolated stylet (his Figure 35 pl. XI). According to the provided scale, the head capsule was 0.62 mm long. The specimen was re-figured in Engel et al. [72].
(6) Gepp [24] re-figured two specimens. The first one was specimen 5401 (his Figure 11a), i.e., the specimen from Gurney [38], but with the modified drawing from Peterson [22] (cited as “Peterson 1960”, apparently a later edition of Peterson [22]). The second one was specimen 5402 (his Figure 11c), i.e., the specimen from Ghilarov [70].
(7) Dorohova [71] re-figured (her Figure 30.2 p. 37) specimen 5402, i.e., the drawing from Ghilarov [70].
(8) Tauber [40] re-figured specimen 5401, i.e., the specimen from Gurney [38], but with the modified drawings from Peterson [22], including the overview (her Figure 33.12 p. 132) and the head in dorsal view (her Figure 33.13 p. 132). Apparently, there are later editions of this contribution also figuring this image (at least 1987, 1991, 2008, our version was labelled 1991). We assume that all of these feature the same images. Hence these also refer to the same specimen.
(9) Monserrat [93] provided several drawings (all on p. 197) of a stage 1 larva of *Dilar pumilus* (specimen 5404). The drawings include the habitus in dorsal view (his Figure 19), the head in dorsal view (his Figure 20), maxilla (his Figure 21), mandible (his Figure 22), and the trunk end in dorsal (his Figure 23) and ventral view (his Figure 24). According to the provided scale, the larva was 6.2 mm long. The specimen was re-figured in Makarkin [61] and Makarkin and Tshistjakov [94].
(10) New [25] re-figured 5402, i.e., the larva from Ghilarov [70]. The images include the habitus in dorsal view (his Figure 14. p. 100) and the head in dorsal (his Figure 14A p. 100) and ventral view (his Figure 14B p. 100).
(11) Minter [95] provided images of several larvae of *Nallachius krooni*. The first specimen was a stage 1 larva (specimen 5405). The images for this specimen include a habitus drawing in dorsal view (his Figure 1 p. 264), and detail drawings of head in dorsal (his Figure 2 p. 264) and ventral view (his Figure 3 p. 264). According to the provided scale, the larva was 1.1 mm long.
  The second specimen was a stage 2 larva (specimen 5406). The images for this specimen include drawings of the head in dorsal (his Figure 4 p. 265) and ventral view (his Figure 5 p. 265). According to the provided scale, the head capsule was 0.18 mm long.
  The third specimen was a stage 3 larva (specimen 5407). The images for this specimen include drawings of the head in dorsal (his Figure 6 p. 265) and ventral view (his Figure 7 p. 265). According to the provided scale, the head capsule was 0.26 mm long.
  Some details were re-figured by Monserrat [96], Aspöck and Aspöck [3], Makarkin and Tshistjakov [94], and Zimmermann et al. [5].
(12) Makarkin [61] re-figured (his Figure 17.3 p. 43) specimen 5404, i.e., the drawing from Monserrat [93].
(13) Tauber et al. [44] re-figured (their Figure 6I p. 793) specimen 5401, i.e., the specimen from Gurney [38], but with the modified drawings from Peterson [22], yet citing Tauber (1991) [40] as the source.
(14) Monserrat [96] depicted micrographs of a stage 1 larva of *Dilar meridionalis*, recorded under transmitted light. The images include an early stage 1 larva (his Figure 5a p. 13), a later stage 1 larva (with a more elongated trunk), details of the head (his Figure 5c p. 13), trunk appendage (his Figure 5d p. 13), antennae (his Figure 6a p. 14), and labium (his Figure 6b p. 14). All of these images may show the same individual at different times, in the same instar (just earlier and later developmental stages), but this aspect remains unclear. We consider only one specimen (specimen 5408) in order to avoid considering the same individual in the same stage twice. Additionally, drawings of the head in dorsal view (his Figure 7a p. 16) and the labium (his Figure 7b p.16) were also provided. These are interpreted as originating from the same specimen as the one from the micrographs of Monserrat [96]. A scale was provided, indicating that the head capsule was 0.14 mm long.
  Monserrat [96] also re-figured some details (labium, his Figure 7c p. 16; antenna, his Figure 7d p. 16) of a specimen from Minter [95]. Drawings of the trunk appendages of a stage 1 larva of *Dilar dissimilis* were also provided (his Figure 8 p. 17); yet, no habitus image of the larva was shown, and hence it could not be further considered here.
(15) Aspöck and Aspöck [3] re-figured (their Figure 93 p. 487) a ventral view on the head of a specimen from Minter [95].
(16) Makarkin and Tshistjakov [94] re-figured specimen 5404 (their Figure 3 p. 47), i.e., a specimen from Monserrat [93] and a specimen (their Figure 4 p. 47) from Minter [95].
(17) Liu et al. [97] provided a photograph of a larva (specimen 5409) of *Nallachius americanus*, in dorsal view (their Figure 1D p. 449). As the specimen was apparently photographed in the field, the developmental stage is unknown. No indication of size was provided.
(18) Engel et al. [72] re-figured (their Figure 3d p. 541) specimen 5403, i.e., a drawing from MacLeod [23]. The figure legend states “*Nallachius americanus* (Coniopterygidae)”, which is likely a misprint of “*Nallachius americanus* (Dilaridae)”.
(19) Zimmermann et al. [5] re-figured (11.4c p. 365) specimen 5405, i.e., a drawing from Minter [95].
(20) Badano et al. [91] provided a photograph of a larva of Dilaridae in dorsal view (their Figure 4ii p. 679). Yet, it is difficult to access details of the mouthparts and the posterior edge of the head capsule from the photograph. Therefore, the specimen could not be further considered for our analysis.
  Short note: Badano et al. [98] reported new morphological details of stage 1 and stage 2 larvae of *Dilar duelli*. The paper was just published after this study was finished and could therefore not be considered in detail here.

### 3.4. Extant Larvae of Osmylidae 

In total, 28 extant specimens of larvae of Osmylidae (specimens 5601–5628) were found in the literature that could be used for our analysis. Since some species names have changed over time (see e.g., discussion in [23] p. 199), we used the names provided by the original authors for historical reference. The specimens were presented in 57 publications, which are listed in the following.

**Figure 16:**
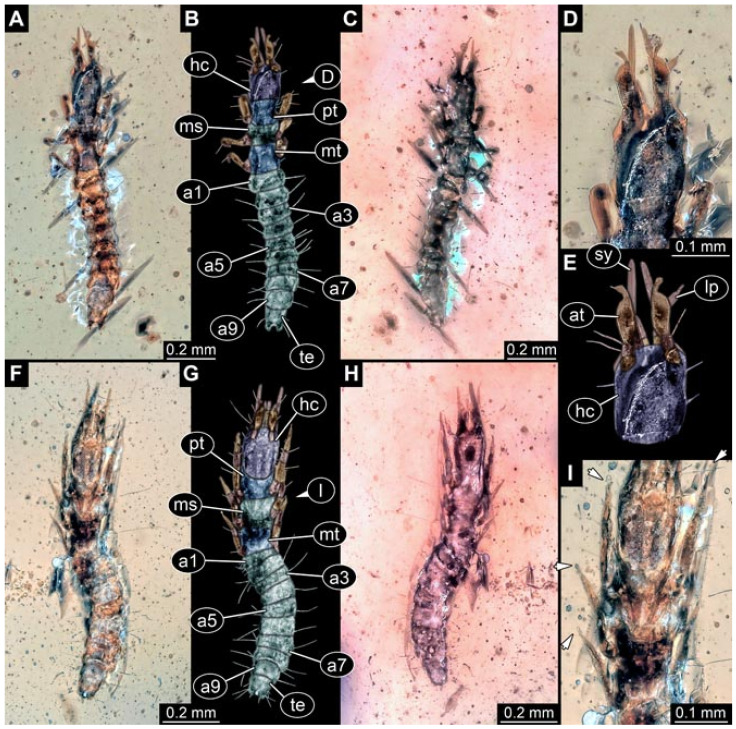
Specimens in Myanmar amber. (**A**–**E**) Specimen 5840 (PED 0900-1). (**A**) Dorsal view. (**B**) Dorsal view, colour-marked. (**C**) Ventral view. (**D**) Close-up of head in dorsal view. (**E**) Close-up of head in dorsal view, colour-marked. (**F**–**I**) Specimen 5841 (PED 0900-2). (**F**) Dorsal view. (**G**) Dorsal view, colour-marked. (**H**) Ventral view. (**I**) Close-up of trunk appendages with empodia (arrows). Abbreviations: a1–a9 = abdomen segment 1–9; at = antenna; hc = head capsule; lp = labial palp; ms = mesothorax; mt = metathorax; pt = prothorax; sy = stylet; te = trunk end.

**Figure 17:**
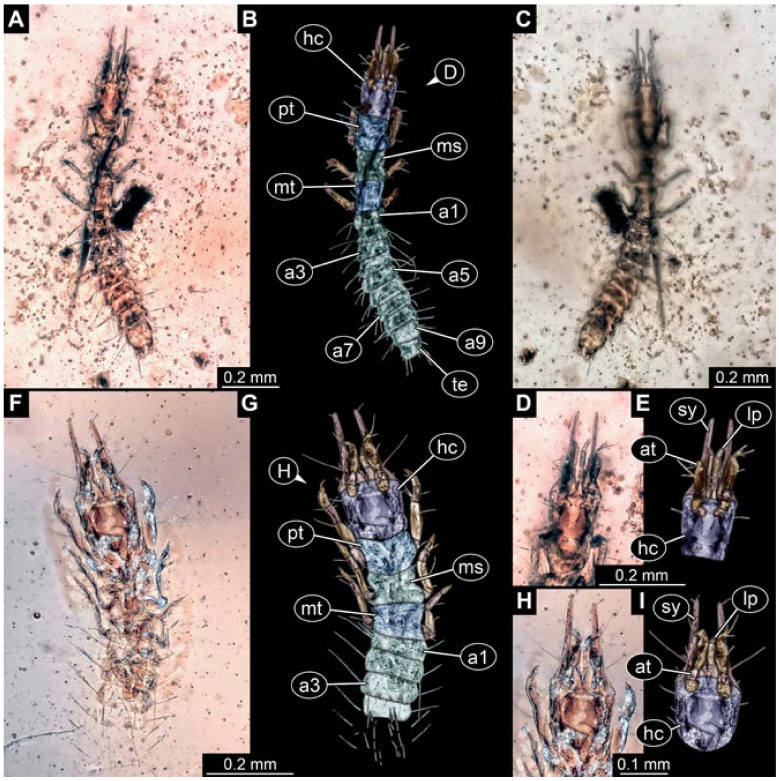
Specimens in Myanmar amber. (**A**–**E**) Specimen 5842 (PED 0900-3). (**A**) Dorsal view. (**B**) Dorsal view, colour-marked. (**C**) Ventral view. (**D**) Close-up of head in dorsal view. (**E**) Close-up of head in dorsal view, colour-marked. (**F**–**I**) Specimen 5843 (PED 0900-4). (**F**) Dorsal view. (**G**) Dorsal view, colour-marked. (**H**) Close-up of head in dorsal view. (**I**) Close-up of head in dorsal view, colour-marked. Abbreviations: a1–a9 = abdomen segment 1–9; at = antenna; hc = head capsule; lp = labial palp; ms = mesothorax; mt = metathorax; pt = prothorax; sy = stylet; te = trunk end.

**Figure 18:**
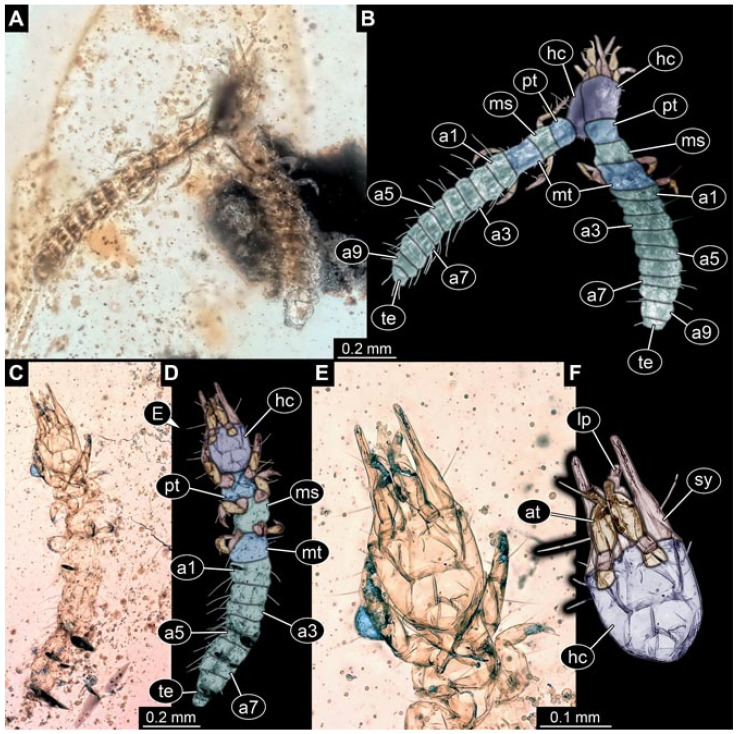
Specimens in Myanmar amber. (**A**–**B**) Specimen 5844 (PED 0900-5, left) and specimen 5845 (PED 0900-6, right). (**A**) Dorsal view. (**B**) Dorsal view, colour-marked. (**C**–**F**) Specimen 5846 (PED 0900-7). (**C**) Ventral view. (**D**) Ventral view, colour-marked. (**E**) Close-up of head in ventral view. (**F**) Close-up of head in ventral view, colour-marked. Abbreviations: a1–a9 = abdomen segment 1–9; at = antenna; hc = head capsule; lp = labial palp; ms = mesothorax; mt = metathorax; pt = prothorax; sy = stylet; te = trunk end.

**Figure 19:**
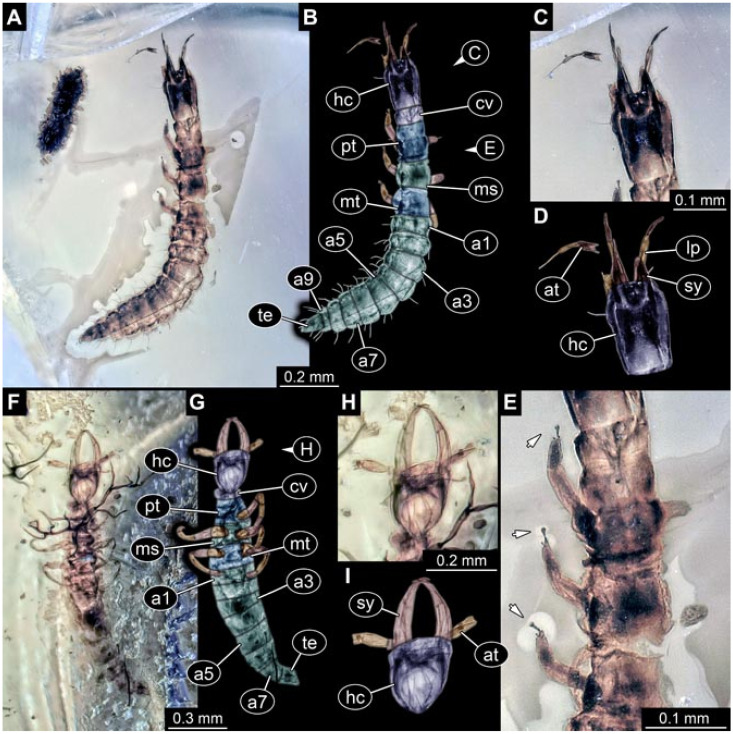
Specimens in Myanmar amber. (**A**–**E**) Specimen 5847 (PED 0769-1). (**A**) Dorsal view. (**B**) Dorsal view, colour-marked. (**C**) Close-up of head in dorsal view. (**D**) Close-up of head in dorsal view, colour-marked. (**E**) Close-up of trunk appendages with empodia (arrows) in dorsal view. (**F**–**I**) Specimen 5848 (PED 0769-2). (**F**) Ventral view. (**G**) Ventral view, colour-marked. (**H**) Close-up of head in ventral view. (**I**) Close-up of head in ventral view, colour-marked. Abbreviations: a1–a9 = abdomen segment 1–9; at = antenna; cv = cervix; hc = head capsule; lp = labial palp; ms = mesothorax; mt = metathorax; pt = prothorax; sy = stylet; te = trunk end.

**Figure 20:**
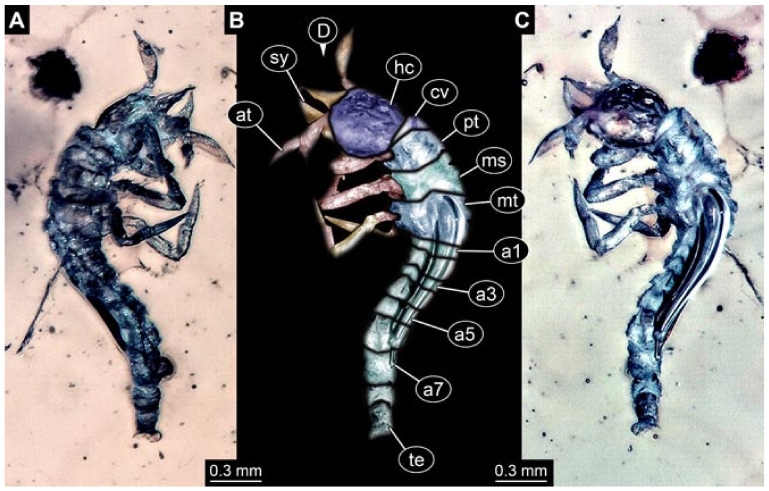
Specimen 5849 (BUB 3711a); Myanmar amber. (**A**) Latero-ventral view. (**B**) Latero-dorsal view, colour-marked. (**C**) Dorsal view. Abbreviations: a1–a7 = abdomen segments 1–7; at = antenna; cv = cervix; hc = head capsule; ms = mesothorax; mt = metathorax; pt = prothorax; sy = stylet; te = trunk end.

**Figure 21:**
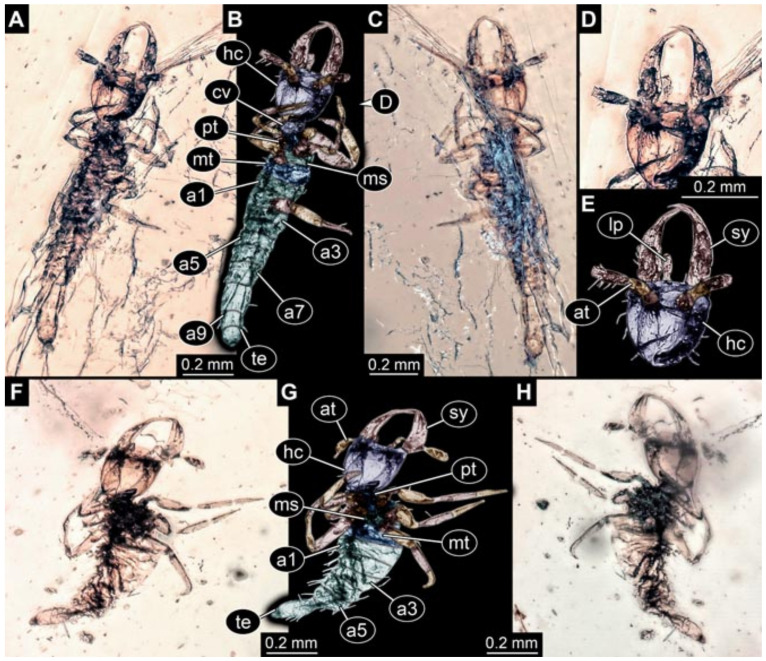
Specimens in Myanmar amber. (**A**–**E**) Specimen 5850 (PED 0791-1). (**A**) Ventral view. (**B**) Ventral view, colour-marked. (**C**) Dorsal view. (**D**) Close-up of head in ventral view. (**E**) Close-up of head in ventral view, colour-marked. (**F**–**H**) Specimen 5851 (PED 0791-2). (**F**) Ventral view. (**G**) Ventral view, colour-marked. (**H**) Dorsal view. Abbreviations: a1–a9 = abdomen segment 1–9; at = antenna; cv = cervix; hc = head capsule; lp = labial palp; ms = mesothorax; mt = metathorax; pt = prothorax; sy = stylet; te = trunk end.

**Figure 22:**
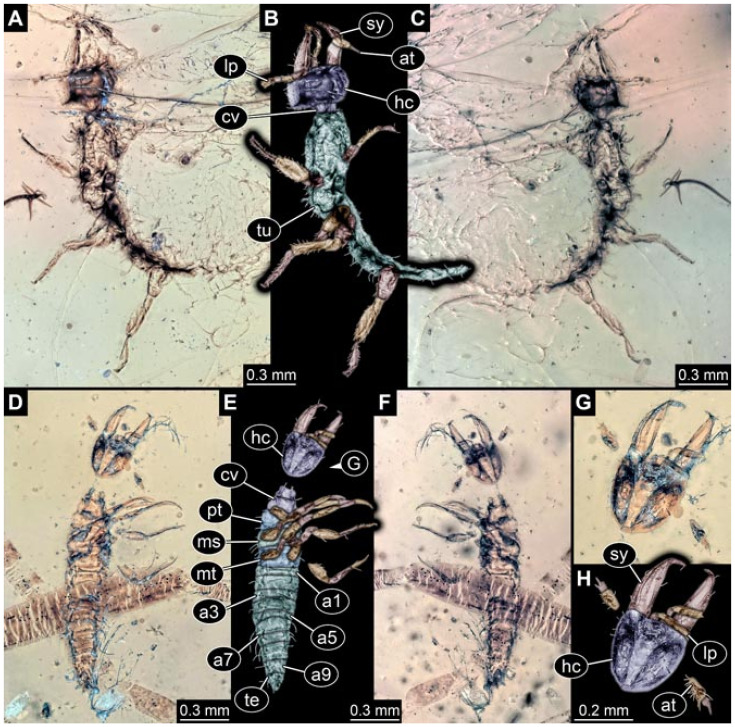
Specimens in Myanmar amber. (**A**–**C**) Specimen 5852 (PED 0791-3). (**A**) Ventral view. (**B**) Ventral view, colour-marked. (**C**) Dorsal view. (**D**–**H**) Specimen 5853 (PED 0791-4). (**D**) Ventral view. (**E**) Ventral view, colour-marked. (**F**) Dorsal view. (**G**) Close-up of head in ventral view. (H) Close-up of head in ventral view, colour-marked. Abbreviations: a1–a9 = abdomen segment 1–9; at = antenna; cv = cervix; hc = head capsule; lp = labial palp; ms = mesothorax; mt = metathorax; pt = prothorax; sy = stylet; te = trunk end; tu = trunk.

**Figure 23:**
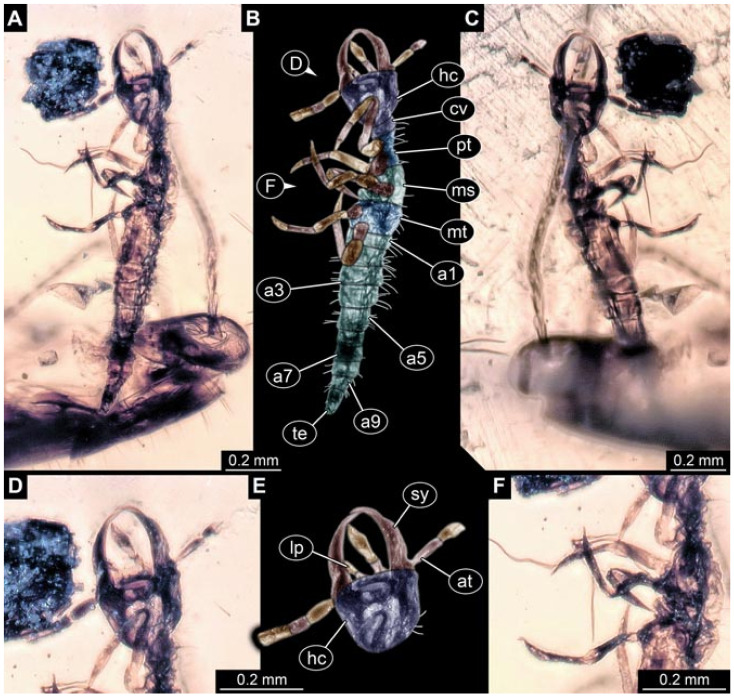
Specimen 5854 (PED 0823); Myanmar amber. (**A**) Ventral view. (**B**) Ventral view, colour-marked. (**C**) Dorsal view. (**D**) Close-up of head in ventral view. (**E**) Close-up of head in ventral view, colour-marked. (**F**) Close-up of trunk appendages. Abbreviations: a1–a9 = abdomen segments 1–9; at = antenna; cv = cervix; hc = head capsule; lp = labial palp; ms = mesothorax; mt = metathorax; pt = prothorax; sy = stylet; te = trunk end.

**Figure 24:**
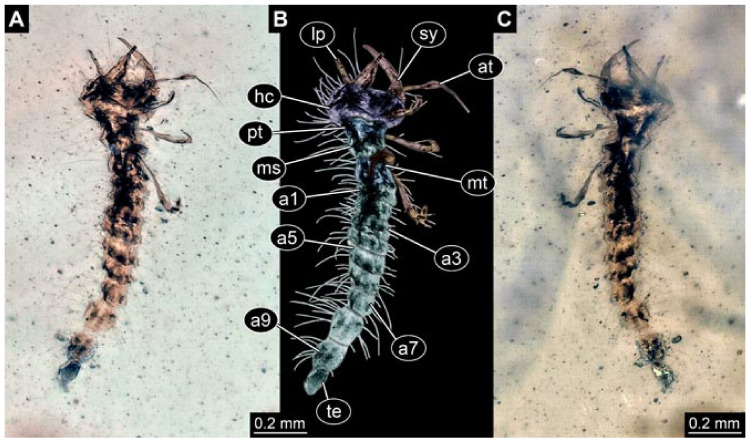
Specimen 5855 (PED 0898); Myanmar amber. (**A**) Dorsal view. (**B**) Dorsal view, colour-marked. (**C**) Ventral view. Abbreviations: a1–a9 = abdomen segments 1–9; at = antenna; hc = head capsule; lp = labial palp; ms = mesothorax; mt = metathorax; pt = prothorax; sy = stylet; te = trunk end.

**Figure 25:**
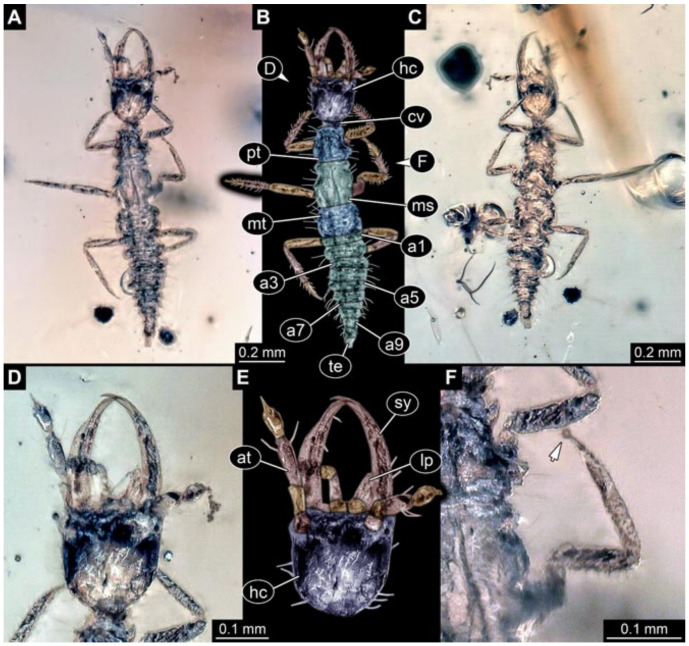
Specimen 5856 (PED 0899); Myanmar amber. (**A**) Dorsal view. (**B**) Dorsal view, colour-marked. (**C**) Ventral view. (**D**) Close-up of head in dorsal view. (**E**) Close-up of head in dorsal view, colour-marked. (**F**) Close-up of trunk appendages with empodia (arrows) in dorsal view. Abbreviations: a1–a9 = abdomen segments 1–9; at = antenna; cv = cervix; hc = head capsule; lp = labial palp; ms = mesothorax; mt = metathorax; pt = prothorax; sy = stylet; te = trunk end.

**Figure 26:**
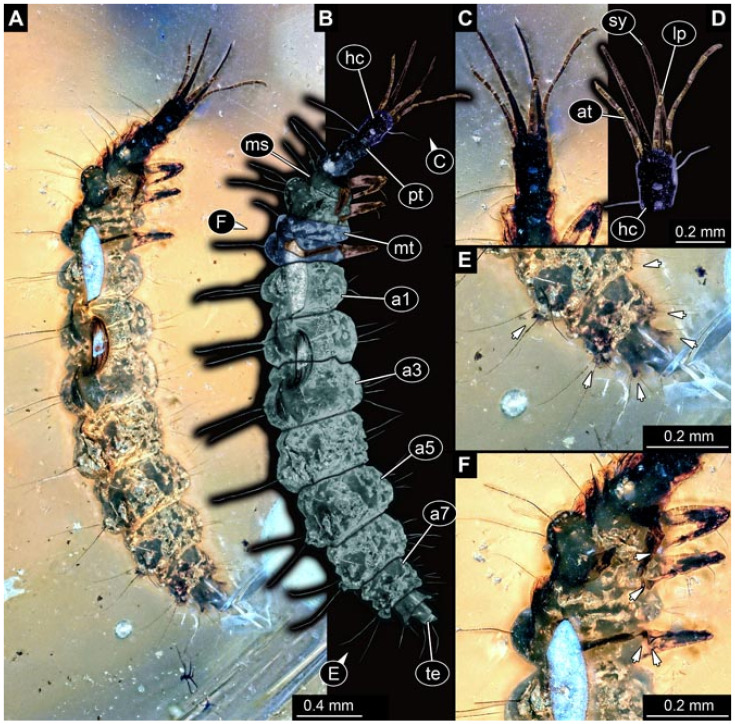
Specimen 5857 (BUB 0049); Myanmar amber. (**A**) Ventral view. (**B**) Ventral view, colour-marked. (**C**) Close-up of head in ventral view. (**D**) Close-up of head in ventral view, colour-marked. (**E**) Close-up of protrusions of abdomen segments (arrows) in ventral view. (**F**) Close up of trunk appendages with empodia (arrows) in ventral view. Abbreviations: a1–a7 = abdomen segments 1–7; at = antenna; hc = head capsule; lp = labial palp; ms = mesothorax; mt = metathorax; pt = prothorax; sy = stylet; te = trunk end.

**Figure 27:**
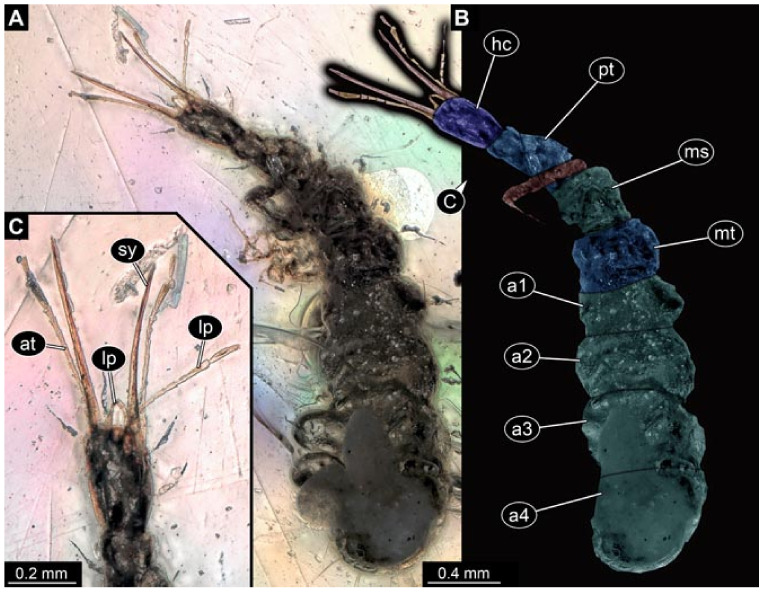
Specimen 5858 (BUB 3368); Myanmar amber. (**A**) Ventral view. (**B**) Ventral view, colour-marked. (**C**) Close-up of head capsule in ventral view. Abbreviations: a1–a4 = abdomen segments 1–4; at = antenna; hc = head capsule; lp = labial palp; ms = mesothorax; mt = metathorax; pt = prothorax; sy = stylet.

**Figure 28:**
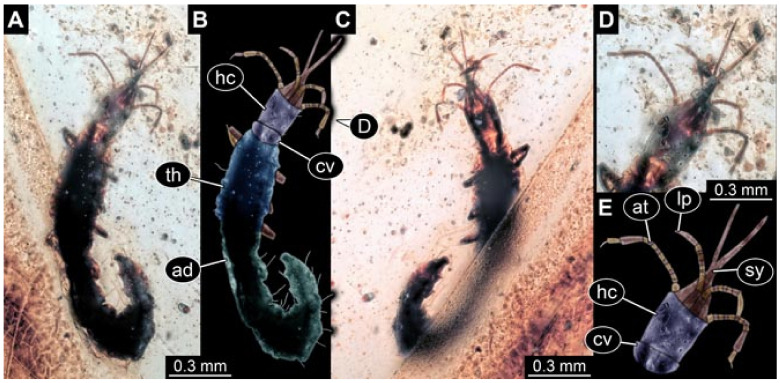
Specimen 5859 (BUB 3737); Myanmar amber. (**A**) Dorsal view. (**B**) Dorsal view, colour-marked. (**C**) Ventral view. (**D**) Close-up of head capsule in dorsal view. (**E**) Close-up of head capsule in dorsal view, colour-marked. Abbreviations: ad = abdomen; at = antenna; cv = cervix; hc = head capsule; lp = labial palp; sy = stylet; th = thorax.

**Figure 29:**
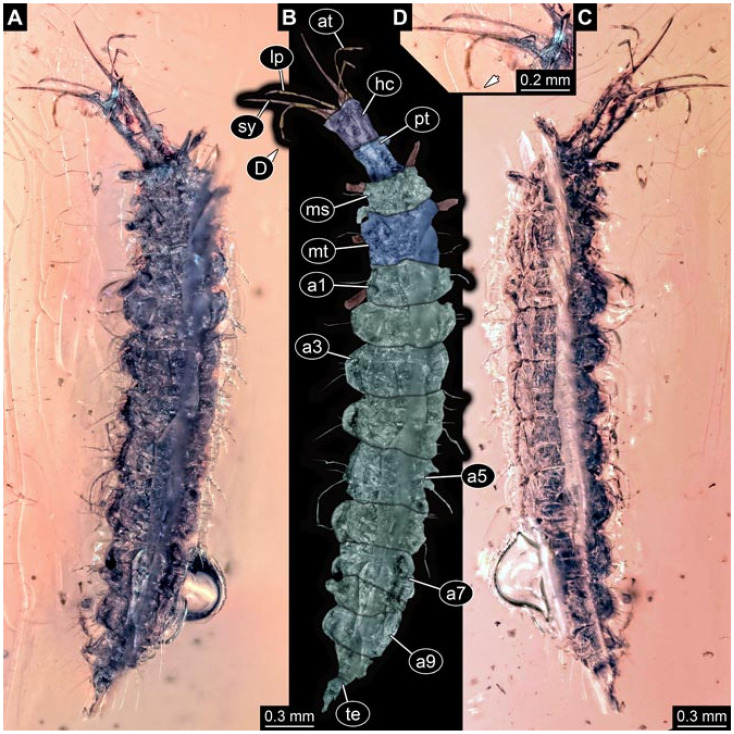
Specimen 5860 (PED 0587); Myanmar amber. (**A**) Dorsal view. (**B**) Dorsal view, colour-marked. (**C**) Ventral view. (**D**) Close-up of antenna with prominent setae (arrow). Abbreviations: a1–a9 = abdomen segments 1–9; at = antenna; hc = head capsule; lp = labial palp; ms = mesothorax; mt = metathorax; pt = prothorax; sy = stylet; te = trunk end.

**Figure 30:**
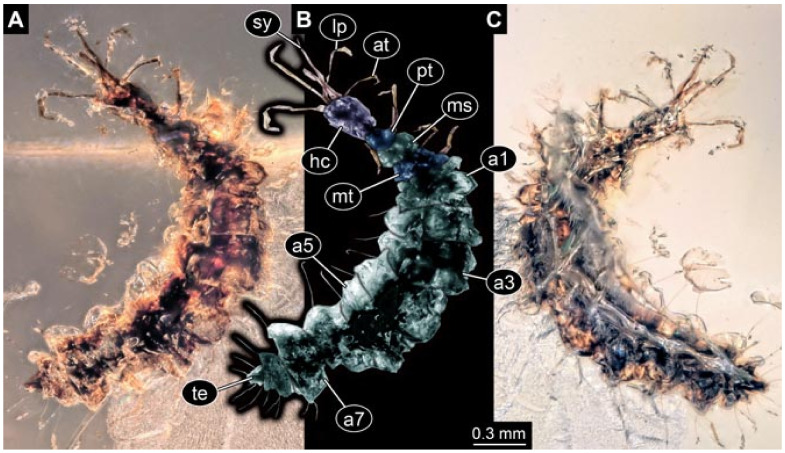
Specimen 5861 (PED 0627); Myanmar amber. (**A**) Dorsal view. (**B**) Dorsal view, colour-marked. (**C**) Ventral view. Abbreviations: a1–a7 = abdomen segments 1–7; at = antenna; hc = head capsule; lp = labial palp; ms = mesothorax; mt = metathorax; pt = prothorax; sy = stylet; te = trunk end.

**Figure 31:**
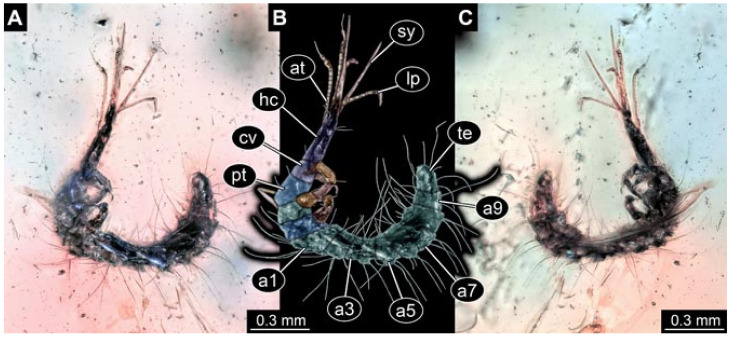
Specimen 5862 (PED 0790); Myanmar amber. (**A**) Dorso-lateral view. (**B**) Dorso-lateral view, colour-marked. (**C**) Ventro-lateral view. Abbreviations: a1–a9 = abdomen segments 1–9; at = antenna; cv = cervix; hc = head capsule; lp = labial palp; pt = prothorax; sy = stylet; te = trunk end.

**Figure 32:**
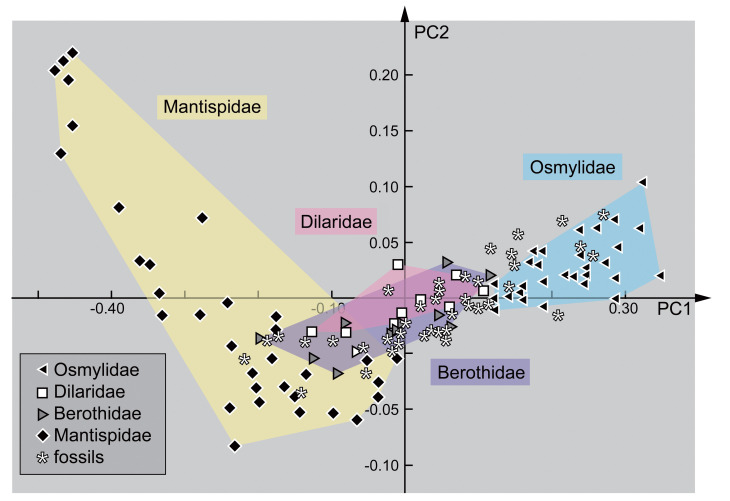
Scatterplot of PC2 vs. PC1 values of head shapes of all specimens.

**Figure 33:**
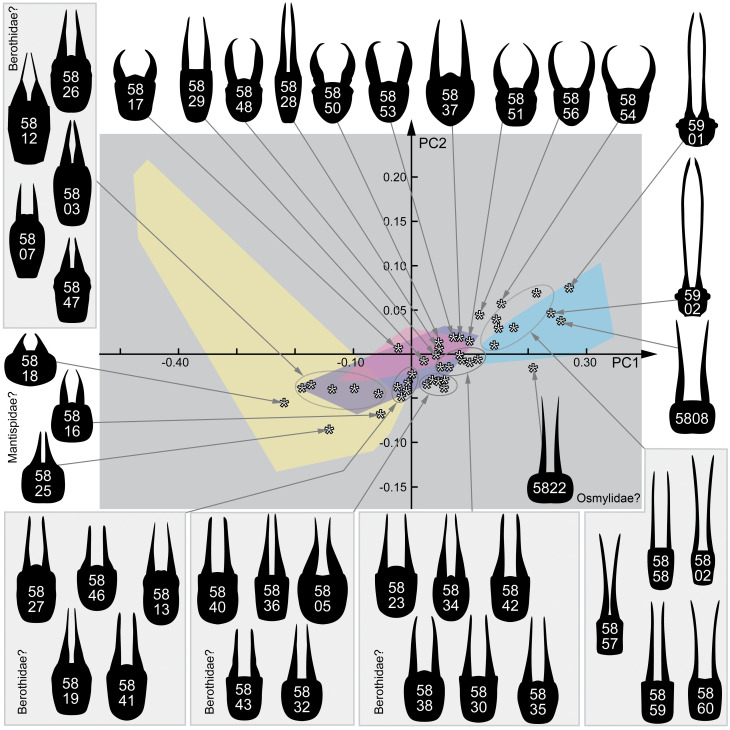
Scatterplot of PC2 vs. PC1 values of head shapes. Occupied areas of extant larvae marked with the same colours as in Figure 32. All fossil specimens are shown with their respective reconstructed outlines. Possible group affinities for some specimens are indicated.

(1) Brauer [99] provided drawings of a larva of *Osmylus* maculatus (specimen 5601). The images include the habitus in dorsal view (his Figure 1a pl. III), the head in lateral view (his Figure 1b pl. III), and the tip of a trunk appendage (his Figure 1c pl. III). No clear indication of size was provided. The specimen was re-figured in Aspöck [100].
(2) Hagen [101] provided several drawings of a larva of *Osmylus maculatus* (specimen 5602). These drawings (all in pl. III) include the habitus in detail in dorsal view (his Figure 9), a partly simplified lateral view (his Figure 10), details of trunk end (his Figures 11 and 13), detail of trunk appendages (his Figure 14), the head in dorsal view (his Figure 15), and a detail of tip of stylet (his Figure 16). There is an unlabelled scale bar on the figure plate, not referring to a specific panel. Therefore, no clear indication of size was provided. The specimen was re-figured in Aspöck [100].
(3) Hudson [102] provided a drawing of a larva (specimen 5603) of *Stenosmylus incisus* in dorsal view (his Figure 3 pl. VIII). No indication of size was provided.
(4) Froggatt [103] provided a drawing of a larva labelled with the name *Porismus strigatus* indicating that it is a larva of Osmylidae. Yet, the larva is clearly a representative of Nymphidae [16,23,104]. Therefore, it could not be further considered here.
(5) Ussing [105] provided a drawing of a larva (specimen 5604) of *Osmylus chrysops* in dorsal view (p. 83). No indication of size was provided.
(6) Lestage [106] provided drawings of two larvae of *Osmylus chrysops.* The first specimen (his Figure 1 p. 227) was a later stage 3 larva in dorsal view (specimen 5605). An unlabelled scale bar is figured next to the drawing. According to the text, the size range of the examined larvae was between 14 mm and 20 mm. The specimen was re-figured by Lestage [8] and Stitz [107].
  The second specimen (his Figure 5 p. 230) was a stage 1 larva (hatchling) in dorsal view (specimen 5606). No indication of size was provided. The specimen was re-figured by Lestage [8], Stitz [54], Jacobs [55], and Haug et al. [7].
(7) Lestage [8] re-figured (his Figure 94 p. 330) specimen 5605, i.e., the larva of *Osmylus chrysops* from Lestage [106].
(8) Withycombe [108] provided a drawing (his Figure 4 pl. XXXVIII) of a larva (specimen 5607) of *Osmylus chrysops.* The size was provided as a magnification factor, which is not informative in combination with the electronic versions available to the present authors. In addition, a ventral view on the head capsule of a later stage larva shortly before pupation (pre-pupa) was provided (his Figure 5 pl. XXXVIII). This second specimen could not be further considered here as it lacks the very long mandibles. The considered specimen was re-figured by Imms [109], Ghilarov [70], Dorohova [71], and Makarkin [61].
(9) Imms [109] presumably re-figured specimen 5607, i.e., the drawing by Withycombe [108]. We could not access the first edition; yet, in the second edition from 1930, the specimen is shown (his Figure 387). The figures are still present in the other editions (published by various publishers; later editions expanded by other authors; e.g., 1924, 1925, 1934; 5th edition 1942; 6th edition 1946, 1948; 8th edition 1951; 9th edition 1957; 9th edition 1960; 9th edition 1964, 1970; 10th edition 1977, 2000, 2017), but with varying figure numbers, e.g., in 10th edition, it is Figure 355 p. 801 [110]).
(10) Withycombe [111] provided details of larvae of *Osmylus chrysops*. This includes a ventral view of the head capsule of a stage 3 larva (his Figure 13 pl. XL) and the tip of the trunk appendages of a stage 1 larva (his Figure 19 pl. XLI). As no complete head is available, none of these could be further considered here.
(11) Tillyard [37] provided a drawing of a larva (specimen 5608) of *Euosmylus stellae* in dorsal view (his Figure U4 p. 310). According to figure legend, the larva was 20 mm long.
(12) Rabaud [112] provided a drawing of a larva (specimen 5609) of Osmylidae in dorsal view (his Figure 5 p. 447) without trunk appendages. The size was provided as a magnification factor, which is uninformative in combination with the electronic versions available to the present authors.
(13) Stitz [107] provided a drawing of a larva of *Osmylus* in dorsal view (his Figure 51 p. XIV 23). Detailed comparison shows a very high similarity to the drawing from Lestage [106]. We therefore consider this a simplified version of specimen 5605.
(14) Stitz [54] provided numerous drawings of larvae of *Osmylops chrysops*. The images include a head in ventral view (his Figure 10 p. 35.79). This is a re-figure of a drawing from Withycombe [111], but simplified, not illustrating the entire stylet length and shape. In the original drawing, the distal part of these is not shown. We could not further consider this drawing.
  The next one was a stage 1 larva in dorsal view (his Figure 110 p. 35.142), which is a re-figure of specimen 5606 (i.e., specimen from Lestage [106]). A later stage larva, also in dorsal view (his Figure 111 p. 35.142), differs from all earlier depictions, especially in the details of the terminal attachment structure (pygopod). The specimen could therefore be further considered for our analysis (specimen 5610). No indication of size was provided. Many details of the larva were provided, including the antenna (his Figure 112 p. 35.143), the tips of maxilla (his Figure 113a p. 35.143), and mandible (his Figure 113b p. 35.143), sections through the stylet (his Figure 114 p. 35.144), a section through the salivary gland (his Figure 115 p. 35.144), the labial palp (his Figure 116 p. 35.144), sections through the palp (his Figure 117 p. 35.144), the anterior body in dorsal view (his Figure 118 p. 35.145), an abdomen sternite (his Figure 119 p. 35.145), excellent details of the terminal end (his Figure 120 p. 35.146), and details of the trunk appendages (his Figure 121 p. 35.147).
(15) Killington [113] apparently wrote about the larva of *Osmylops fulvicephalus*. Yet, we were unable to get a hold of a copy of this work and can therefore not state whether or not it contains any depictions.
(16) David [114] provided drawings of larvae of *Osmylus chrysops.* This included a larva that was still inside the egg, but close to hatching (his Figure 4 p. 153) and a stage 3 larva in dorsal view (his Figure 5 p. 155). Yet, these drawings appear to be strongly simplified and could not be further considered here as they do not provide sufficient details.
(17) Killington [115] figured a drawing of a stage 3 larva (specimen 5611) of *Osmylus fulvicephalus* in dorsal view (his Figure 1 pl. IX). The figure shows a small empodium, also mentioned in the text, which is often not shown in drawings of larvae of Osmylidae. According to the text and the figure legend, the larva was 15 mm long. The specimen was re-figured by Parfin and Gurney [116], Kimmins [117], and Aspöck and Aspöck [60].
(18) Genay [118] provided two drawings of a larva of *Osmylus chrysops*. The images include the head capsule in dorsal and ventral view (his Figure 8 upper and lower between pages 22 and 23). As the distal parts of the mouthparts are missing in both views, the specimen could not be further considered here.
(19) Parfin and Gurney [116] re-figured (their Figure 3C p. 431) specimen 5611, i.e., the one from Killington [115].
(20) Kawashima [119] provided several drawings of a larva (specimen 5612) of *Spilosmylus flavicornis*. These drawings included (all on pl. 2) the habitus in dorsal view (his Figure A), the head capsule in dorsal (his Figure B) and ventral view (his Figure C), labial palp (his Figure D), antenna (his Figure E), and a trunk appendage (his Figure F). According to the text, the larva was 9 mm long.
(21) Fraser [120] provided a drawing (his Figure 14g p. 33) of a larva (specimen 5613) of *Osmylus fulvicaphalus* in dorsal view. No indication of size was provided.
(22) Wundt [121] provided numerous images of a later stage larva of Osmylus chrysops. The images include a photograph of an entire larva in dorsal view (his Figure 1 p. 558) as well as drawings of the head in dorsal (his Figure 2a p. 561) and ventral view (his Figure 2b p. 561), of details of the head capsule (his Figure 3 p. 563, his Figure 33 p. 641, his Figure 34 p. 642), the mandibles (his Figure 4 p. 569, his Figure 5 p. 570, his Figure 6 p. 573, his Figure 7 p. 575, his Figure 8 p. 576), the maxillae (his Figure 9 p. 578, his Figure 10 p. 583, his Figure 11 p. 585, his Figure 12 p. 586, his Figure 13 p. 587), anterior nervous system (his Figure 14 p. 588, his Figure 22 p. 612, his Figure 28 p. 634), the labium (his Figure 15 p. 592, his Figure 17 p. 595, his Figure 18 p. 597), the salivarium (his Figure 16 p. 593), pre-oral chamber (his Figure 19 p. 601, his Figure 23 p. 615, his Figure 25 pp. 622–623), musculature of the head capsule (his Figure 20 p. 604, his Figure 21 p. 609), the antenna (his Figure 26 p. 627, his Figure 27 p. 629), specialised inner organs (his Figure 29 p. 635, his Figure 30 p. 636, his Figure 31 p. 637, his Figure 32 p. 639), and trachea (his Figure 35 p. 644). The text states that stage 2 and 3 larvae were used. We consider a single specimen (specimen 5614) for our analysis, as represented by the image of the head (his Figure 2a p. 561). According to the provided scale, the head capsule was 1.4 mm long. The specimen was re-figured in Aspöck and Aspöck [3].
(23) Ghilarov [70] re-figured (his Figure 11 p. 410) specimen 5607, i.e., the specimen from Withycombe [108], yet without reference to the source.
(24) Kimmins [117] re-figured (his Figure 7A p. 9) specimen 5611, i.e., the drawing from Killington [115].
(25) Aspöck and Aspöck [60] re-figured (their Figure 55 p. 266) specimen 5611, i.e., the specimen from Killington [115].
(26) MacLeod [23] provided several drawings of a larva of *Kempynus* (specimen 5615). The images include the head in ventral (his Figure 21 pl. VII), dorsal (his Figure 22 pl. VII), and lateral view (his Figure 23 pl. VIII), inner arrangement of the head capsule (his Figure 24 pl. VIII), and details of the mandible (his Figure 25 pl. VIII). According to the provided scale, the head capsule was 1.4 mm long.
(27) Ward [122] wrote about the larva of *Osmylops fulvicephalus*. Yet, we were unable to acquire a copy of this work and can therefore not state whether or not it contains any depictions.
(28) Riek [1] provided drawings of two larvae of Osmylidae. The first showed a head of a larva of *Porismus strigatus* in ventral view (his Figure 29.5A). Yet, the stylets are not fully shown, and hence this specimen could not be further considered here.
  The second one showed a larva of *Kempynus* (specimen 5616) in dorsal view (his Figure 29.10E p. 486), accompanied by detail of the head in dorsal view (his Figure 29.10F p. 486). The stage of the larva remains unclear. No indication of size was provided. The specimen was re-figured by New [25,43,123,124], Tauber et al. [44], and Heckman [51].
(29) New [125] provided drawings of details of a larva of *Stenosmylus tenuis*. These drawings include the labium (his Figure 5 p. 26), antenna (his Figure 6 p. 26), trunk appendages (his Figure 7 p. 26), and the anchoring structure at the terminal end (his Figure 8 p. 26). As no image of the head was provided, the specimen could not be further considered here.
(30) Gepp [24] provided drawings of a stage 3 larva of *Osmylus fulvicephalus* (specimen 5617). The images include the habitus in dorsal view (his Figure 10a pl. 5 p. 196) and a dorsal view of the head (his Figure 10b pl. 5 p. 196). According to figure legend, the larva was 17 mm long.
(31) Dorohova [71] provided a drawing of a larva of *Osmylus fulvicephalus* (her Figure 38 p. 47). The drawing resembles the drawing of Withycombe [108] in most aspects, but shows some differences in the positions of the trunk appendages. Yet, as the entire body outline and the majority of setae match, we consider this drawing as a re-figure of specimen 5607.
(32) New [25] re-figured (his Figure 137 p. 98) specimen 5616, i.e., the drawings of the larva of *Kempynus* from Riek [1].
(33) New [43] apparently re-figured specimen 5616, i.e., the specimen from Riek [1]. This source was not directly seen by the current authors and the information is based on a reference in Tauber et al. [44]. Therefore, the figure number remains unknown.
(34) New [123] provided a simplified drawing (his Figure 10 p. 32) of specimen 5616, i.e., the specimen from Riek [1], but without reference.
(35) Makarkin [61] provided a drawing of a larva of *Osmylus fulvicephalus* (his Figure 16.4 p. 42). The drawing resembles the drawing of Withycombe [108] in most aspects, but shows some differences in the positions of the trunk appendages. Yet, as the entire body outline and the majority of setae match, we consider this drawing as a re-figure of specimen 5607.
(36) Jacobs [55] re-figured (his Figure O-30) drawings of two specimens, the specimens shown in Stitz [54]. The one specimen is from Withycombe [111] (but the amended version from Stitz [54]), and the other is specimen 5606, i.e., the one from Lestage [106].
(37) Aspöck and Aspöck [2] provided a photograph (their Figure 46, p. 19) of a larva (specimen 5618) of *Osmylus fulvicephalus* in dorsal view. According to the figure legend, the larva was 16 mm long. The larval stage remains unclear. The photograph was recorded by Peter Duelli. The specimen was re-figured in Aspöck [126], Grimaldi and Engel [127], and Aspöck and Aspöck [3].
(38) Gepp [128] provided a photograph (his Figure 22 p. 194) of a larva (specimen 5619) of *Osmylus fulvicephalus* in dorsal view. According to figure legend, the larva was 13 mm long. The larval stage remains unclear.
(39) Aspöck [100] re-figured some of the historically important images. These included specimen 5601 (his Figure 43 p. 30), i.e., the specimen from Brauer [99], and specimen 5602 (his Figure 54 p. 31), i.e., the specimen from Hagen [101] (entire plate).
(40) Aspöck [126] re-figured specimen 5618, i.e., the specimen from Aspöck and Aspöck [2]. The source was cited as Aspöck and Aspöck (1991), but the reference was neither available in the reference list, nor in the literature.
(41) Gepp [129] provided numerous images of larvae of *Osmylus fulvicephalus*. The first one was a photograph of stage 1 larvae (in varying states of feeding) in dorsal view (his Figure 4 p. 327). Yet, these images are a bit too small to provide enough detail and could not be further considered here.
  The second one was a drawing of a late stage 1 larva (specimen 5620) in dorsal view (his Figure 5 p. 327). This drawing provides excellent details. According to figure legend, the larva was 5.6 mm long.
  Further images show many aspects of a stage 3 larva, possibly of a single specimen. The images include a micrograph in dorsal view (his Figure 6 p. 328), a drawing in dorso-lateral view (his Figure 7 p. 329) and in lateral view (his Figure 8 p. 329), details of the tergites (his Figure 9 p. 330), the trunk appendages (his Figure 10 p. 330), the head in ventral view (his Figure 11 p. 331), and a schematic lateral view of the head (his Figure 12 p. 331). No clear indication of size was provided for the dorsal view. The micrograph of the habitus shows a large similarity to specimen 5619, i.e., the specimen from Gepp [128] with only the position of the trunk appendages being slightly different. We still consider this as additional images of specimen 5619 in order to avoid considering the same specimen twice.
(42) Tauber et al. [44] re-figured specimen 5616 (their Figure 6D p. 793), i.e., the specimen from Riek [1], citing New [43] as the source.
(43) New [124] re-figured specimen 5616 (his Figure 3E,F p. 496), i.e., the specimen from Riek [1], citing New [43] as the source.
(44) Grimaldi and Engel [127] re-figured specimen 5618 (their Figure 9.26 p. 349), i.e., the specimen from Aspöck and Aspöck [2].
(45) Aspöck and Aspöck [3] provided a photograph of a larva (specimen 5621) of *Osmylus fulvicephalus* in dorsal view (their Figure 33 p. 464). According to the figure legend, the larva was 16 mm long. The larval stage remains unclear. The photograph was recorded by Franziska Anderle.
  Aspöck and Aspöck [3] also re-figured two other specimens. This included specimen 5614 (their Figure 69 p. 477; their Figure 90 p. 487), i.e., the specimen from Wundt [121] and specimen 5618 (their Figure 78 p. 483), i.e., the specimen from Aspöck and Aspöck [2].
(46) Cover and Bogan [130] provided photographs of a stage 3 larva of *Osmylus fulvicephalus* in lateral (their Figure 41.6a p. 1066) and dorso-lateral view (their Figure 41.6b p. 1066). As the head is not well accessible in dorsal view, the specimen could not be further considered here.
(47) Matsuno and Yoshitomi [131] provided images of several stage 3 larvae of Osmylidae. In total, the larvae of three species were examined: *Osmylus hyalinatus*, *O. pryeri* and *O. tesselatus*.
  The larva of *Osmylus hyalinatus* (specimen 5622) was provided as a photograph in the field (their Figure 36) and a number of details as drawings including the head in dorsal view (their Figure 15), the anterior edge of the head (their Figure 5), the tip of the antenna (their Figure 8) and the labial palp (their Figure 6), the entire antennae (their Figure 7), setae arrangement of the thorax in dorsal (their Figure 9 left, 18), ventral (their Figure 9 right), and lateral view (their Figure 10), a single thorax sclerite (their Figure 19), setae arrangement of the abdomen in dorsal (their Figures 11 and 24) and ventral view (their Figure 12), ventral details of abdomen segment 7 (their Figure 27), dorsal (their Figure 30) and ventral (their Figure 31) details of abdomen segment 9, and the trunk end in dorsal (their Figure 13) and ventral view (their Figure 14). According to the provided scale, the head capsule was 1 mm long.
  The larva of *Osmylus pryeri* (specimen 5623) was provided as a photograph in the field (their Figure 37) in addition to a number of details as drawings, including the head in dorsal (their Figures 1 and 16), ventral (their Figure 2), and lateral view (their Figure 3), the anterior edge of the head (their Figure 4), setae arrangement of the thorax in dorsal view (their Figure 20), a single thorax sclerite (their Figure 21), setae arrangement of the abdomen in dorsal view (their Figure 25), ventral details of abdomen segment 7 (their Figure 28), and abdomen segment 9 in dorsal (their Figure 32) and ventral view (their Figure 33). According to the provided scale, the head capsule was 1.3 mm long.
  The larva of *Osmylus tesselatus* (specimen 5624) was provided as a photograph in the field (their Figure 37) and a number of details as drawings, including the head in dorsal view (their Figure 17), setae arrangement of the thorax in dorsal (their Figure 22), a single thorax sclerite (their Figure 23), setae arrangement of the abdomen in dorsal (their Figure 26), ventral details of abdomen segment 7 (their Figure 29), and abdomen segment 9 in dorsal (their Figure 34) and ventral view (their Figure 35). According to the provided scale, the head capsule was 1.4 mm long.
(48) Glime [132] figured several photographs of larvae of Osmylidae in the field. The first (her Figure 2 p. 11-8-2) was an image of a larva of Osmylus fulvicephalus in postero-dorsal view. The second (her Figure 5 p. 11-8-3) was a close up of the head region in antero-dorsal view of a similar larva. The third (her Figure 6 p 11-8-3) was an image of a larva of *Kempynus* in lateral view. All images do not provide direct access to the head in dorsal orientation and could therefore not be further considered here.
(49) Glime [133] re-figured (her Figure 7 p. 12-8-3) a specimen from Glime [132].
(50) Heckman [51] re-figured (his Figure 2.28 p. 65) the simplified drawing from New [123] (also cited as that), i.e., the specimen from Riek [1].
(51) Matsuno [134] provided images of at least one larva *Osmylus hyalinatus* (specimen 5625). Images show a larva in “crumpled” state (his Figure 2A p. 3) and one in an “inflated” state (his Figure 2D p. 3). It remains unclear whether these two images show the same individual. To avoid considering the same specimen twice, we only considered one of the images, namely that in the “inflated” state. No indication of size was provided.
(52) Winterton et al. [135] provided photographs of several larvae in the field. These included larvae of *Kempynus* (their Figure 2C p. 3; recorded by Kristi Ellington), Isostenosmylus (their Figure 2D p. 3; recorded by Enio Branco), and Stenosmylinae (their Figure 2E p. 3 recorded by Kristi Ellington). While the images are quite detailed and could be used for other types of analyses, the heads are not ideally accessible in dorsal view or provide only a low contrast of stylets against the background. Hence, none of these specimens could be further considered.
(53) Martins et al. [136] provided detailed images of several larvae of Osmylidae. These included images of larvae of *Kempynus* and *Isostenosmylus pulverulentus*.
  The images of *Kempynus* included photographs of an anterior body region of a stage 3 larva in lateral view (their Figure 2 upper p. 5) and a stage 2 larva in lateral view (their Figure 2 lower p. 5) as well as photographs of stage 3 larvae in the field (their Figure 3g,h p. 6) and drawings of stage 3 larvae of the head in dorsal (their Figure 4a p. 7), ventral (their Figure 4b p. 7), and lateral view (their Figure 4c p. 7), of the antenna (their Figure 5a p. 8), the tip of the antenna (their Figure 5b p. 8), anterior edge of the head (their Figure 5c p. 8), the tip of the labial palp (their Figure 5d p. 8), and the trunk appendages (their Figure 5e,f p. 8), the setae arrangement of the thorax in dorsal (their Figure 6a p. 10), ventral (their Figure 6b p. 10), and lateral view (their Figure 6c p. 10), the setae arrangement on the abdomen segments in dorsal (their Figure 7a p. 12) and ventral view (their Figure 7b p. 12), and the setae arrangement on the trunk end in dorsal (their Figure 8a p. 13) and ventral view (their Figure 8b p. 13). We here considered the drawings of the head as a single specimen (specimen 5626).
  Images of *Isostenosmylus pulverulentus* included photographs of an anterior body region of a stage 3 larva in lateral view (their Figure 9 upper p. 15) and a stage 2 larva in lateral view (their Figure 9 lower p. 15), drawings of stage 3 larvae with the head in dorsal (their Figure 10a p. 16), ventral (their Figure 10b p. 16), and lateral view (their Figure 10c p. 16), of the antenna (their Figure 11a p. 17), the tip of the antenna (their Figure 11b p. 17), anterior edge of the head (their Figure 11c p. 17), the tip of the labial palp (their Figure 11d p. 17), and the trunk appendages (their Figure 11e,f p. 17), the setae arrangement of the thorax in dorsal (their Figure 12a p. 19), ventral (their Figure 12b p. 19), and lateral view (their Figure 12c p. 19), the setae arrangement on the abdomen segments in dorsal (their Figure 13a p. 21) and ventral view (their Figure 13b p. 21), and the setae arrangement on the trunk end in dorsal (their Figure 14a p. 22) and ventral view (their Figure 14b p. 22). We here considered the drawings of the head as a single specimen (specimen 5627).
(54) Haug et al. [7] re-figured specimen 5606 (their Figure 5C p. 11), i.e., a specimen from Lestage [106] (but cited Stitz [54] as the source).
(55) Winterton et al. [10] re-figured the three photographs (their Figure 1 p. 5) from Winterton et al. [135].
(56) Zimmermann et al. [5] re-figured specimen 5614 (their Figure 11.4a p. 365), i.e., a drawing from Wundt [121].
(57) Badano et al. [91] provided a photograph of a larva (specimen 5628) of Osmylidae in dorsal view (their Figure 4i p. 679). No indication of size was provided.

### 3.5. Fossil Lacewing Larvae with Straight or Almost Straight Stylets from the Literature

In total, 23 fossil specimens of lacewing larvae with straight or almost straight stylets (specimens 5801–5821, 5901, 5902) were found in the literature. These were presented in 16 publications, which are listed as follows.

(1) Whalley [137] provided images of a larva of Berothidae (specimen 5801) preserved in Lebanese amber. This included micrographs of the habitus in more less-dorsal view (his Figure 9 p. 163) and a close-up of the head (his Figure 10 p. 163). No indication of size was provided. The details are not sufficient to further consider the specimen for our analysis.
(2) Grimaldi et al. [138] provided a micrograph (their Figure 28f p. 44) of a larva (specimen 5802) of Osmylidae preserved in Myanmar amber. The specimen is part of the collection of the American Museum of Natural History, New York, USA (AMNH Bu-267). The image is rather small, yet more detailed images were provided in Engel and Grimaldi [139] (see there for details).
(3) Janzen [140] provided a detailed drawing of a larva of Berothidae preserved in Baltic amber in dorsal view (his Figure 58 p. 120). According to the figure legend, the larva (specimen 5803) was 5.4 mm long. The specimen is part of the collection of Hoffeins (CCHH 1270-1, now SDEI Lep-103564). Originally the specimen was interpreted as a larva of Chrysopidae, but later re-interpreted as a larva of Berothidae [13]. The specimen was re-documented for the present study (Figure 1).
  Janzen [140] also figured a micrograph of a small larva (specimen 5804) attached to a spider (his Figures 107 and 108 p. 81). He interpreted this as the larva of a beetle. Later, the specimen was interpreted as a larva of Mantispidae [11]. Yet, especially the head region is not accessible, and hence the specimen could not be considered for our analysis. The specimen was re-figured by Wunderlich [141], Ohl [11], and Haug et al. [12].
(4) Wunderlich [141] re-figured specimen 5804 (his photo 605 p. 559), i.e., the larva attached to a spider from Janzen [140].
(5) Engel and Grimaldi [139] provided numerous fossil larvae with straight stylets originating from Cretaceous amber.
  The first specimen was a small larva (specimen 5805) preserved in amber from Myanmar. Images include a micrograph of the habitus (their Figure 7 p. 54) and a drawing (their Figure 8 p. 54). According to the provided scale, the specimen was 0.6 mm long. The specimen is part of the collection of the American Museum of Natural History, New York, USA (AMNH Bu-126). Originally, the specimen was interpreted as a possible larva of Psychopsidae. Later it was re-interpreted as a possible larva of Berothidae [21] (see also discussion in [15]). According to Pérez-de la Fuente et al. [21] (supplement), there is a second, similar-appearing specimen, yet it appears not to have been figured.
  The second specimen was a re-figure of specimen 5802, i.e., the larva of Osmylidae preserved in amber from Myanmar, already published in Grimaldi et al. [138]. Images included micrographs of the entire larva in dorsal view (their Figure 9 p. 55) and a close-up on the head (their Figure 10 p. 55) with drawings of the same aspects (their Figure 11 p. 56). It is important to note that at least the first trunk appendage appears to bear an empodium (see further below). According to the provided scale, the larva was 4.5 mm long.
  The third specimen was a larva hatching from the egg, preserved in Canadian amber (specimen 5806). Images included micrographs of the habitus (their Figures 12 and 13 p. 57) and a drawing of the habitus (their Figure 14 p. 58). According to the provided scale, the larva was 0.8 mm long. The specimen is part of the amber collection of the Canadian National Collection, Ottawa, Canada (CAS-1096). The specimen was originally interpreted as a larva of Chrysopidae, but was later interpreted as a representative of Berothidae [13] (p. 250). The interpretation is supported by Pérez-de la Fuente et al. [21]. The details of the stylets are not easy to interpret, and therefore the specimen could not be further considered for the analysis performed here.
  The fourth specimen was a larva (specimen 5807) of Berothidae preserved in amber from Myanmar. Images include a micrograph of the entire larva in ventral view (their Figure 42 p. 75) and a drawing of the head in ventral view (their Figure 43 p. 75). According to the provided scale, the head capsule was 0.6 mm long. The specimen is part of the collection of the American Museum of Natural History, New York, USA (AMNH Bu-1297).
(6) Wichard et al. [142] provided three images of a small larva (specimen 5808) of Osmylidae preserved in Baltic amber. This included a micrograph of the habitus in ventral view (their Figure 07.04a p. 88), a detail of the head region in dorsal view (their Figure 07.04b p. 88), and a drawing of an antenna (their Figure 07.04c p. 88). According to the provided scale, the larva was 1.5 mm long.
(7) Ohl [11] re-figured the specimen from Janzen [140] and interpreted it as a larva of Mantispidae.
(8) Wunderlich [83] reported three stage 1 larvae of Mantispidae (specimens 5809–5811) preserved in a single piece of Baltic amber. The larvae were preserved in close association with a male spider, two of them even in direct contact to it. The larvae were stated to be 7 mm, 9 mm, and 10 mm long, respectively. This would be extremely large for stage 1 larvae of mantis lacewings. The images include micrographs of the spider in overview (photo 35 p. 344) and close-ups of two larvae (photo 36 p. 344). Yet, neither are accessible in dorsal view and could therefore not be further considered for our analysis. The specimens are part of the collection of Jörg Wunderlich, F2275/BB/AR/CJW (this contribution had been overlooked by Nagler and Haug [85] and Haug et al. [12]).
(9) Wedmann et al. [13] provided five fossil larvae of the group Berothidae, all originating from Baltic amber.
  The first specimen (specimen 5812) was provided with numerous images including a micrograph (their Figure 1A p. 238) and a drawing (their Figure 1B p. 238) in dorsal view, micrographs of the head in dorsal (their Figure 2A p. 239) and ventral view (their Figure 2B p. 239), drawings of the head in dorsal (their Figure 2C p. 239) and ventral view (their Figure 2D p. 239), and a drawing of a trunk appendage (their Figure 2E p. 239). According to the text, the larva was 1.83 mm long. The specimen is part of the collection of Thomas Weiterschan (No. 240).
  The second specimen (specimen 5813) was provided with numerous images including a micrograph (their Figure 3A p. 240) and a drawing (their Figure 3B p. 240) in dorsal view, micrographs of the head in dorsal (their Figure 4A p. 241) and ventral view (their Figure 4B p. 241), a micrograph of the trunk end (their Figure 4C p. 241), drawings of the head in dorsal (their Figure 5A p. 242) and ventral view (their Figure 5B p. 242), a drawing of a trunk appendage (their Figure 5C p. 242), and a drawing of the trunk end (their Figure 5D p. 242). According to the text, the larva was 2.25 mm long. The specimen is part of the collection of Thomas Weiterschan (No. 1555).
  The third specimen is specimen 5803, the same specimen as in Janzen [140], but with more detailed images, including a micrograph of the habitus in dorsal view (their Figure 6 p. 244), a micrograph of the head in dorsal view (their Figure 7A p. 244), a drawing of the head in dorsal view (their Figure 7B p. 244), a drawing of the labial palp (their Figure 7C p. 244), and a drawing of a trunk appendage (their Figure 7D p. 244).
  The fourth specimen (specimen 5814) was also provided with numerous images, including micrographs of the left and right body side (their Figure 8A,B p. 246), drawings of one body side (their Figure 9A p. 247), the head in latero-ventral view (their Figure 9B p. 247), a close-up of the eye region (their Figure 9C p. 247), and volume renderings of a SRµCT scan of the head in dorsal view (their Figure 10A p. 248), the stylets (their Figure 10B p. 248) and the entire specimen revealing some inner structures (their Figure 10C p. 248). According to the text, the larva was 3.1 mm long. The specimen is part of the collection of the Senckenberg Museum, Frankfurt, Germany (SMF Be 1297). Despite the detailed documentation and re-documentation of the specimen for the present study (Figure 2), there is no view that allows a reconstruction of the outline of the head in dorsal view with the stylets in forward orientation. Therefore, the specimen could unfortunately not be further considered for the analysis.
  The fifth specimen (specimen 5815) was figured as micrographs of the habitus (their Figure 11A p. 249) and as a close-up of the head (their Figure 11B p. 249). According to the text, the larva was 7.5 mm long. It is part of the collection of Thomas Schäfer. It was only briefly discussed and the orientation of the specimen does not allow us to consider the specimen further for the analysis performed herein.
(10) Xia et al. [143] provided an image of a larva with very long stylets preserved in Myanmar amber (upper image p. 95). Pérez-de la Fuente et al. [21] suggested that this larva could be a representative of Osmylidae. Yet, the stylets appear quite curved inwards; therefore, the larva appears rather different from lance lacewing larvae. Due to this uncertainty, we could not consider this larva further here.
(11) Engel [144] provided images of a larva (specimen 5816) preserved in amber from Myanmar. Images include micrographs of an overview of the amber piece showing syn-inclusions (his Figure 9 p. 11) and the habitus in ventral view (his Figure 10 p. 11). According to the text, the larva was 1.5 mm long. The specimen is part of the collection of the American Museum of Natural History, New York, USA (AMNH Bu-275). Originally, the specimen was interpreted as a possible larva of Coniopterygidae, but later interpreted as a larva of Berothidae [21].
(12) Zhang [145] provided a micrograph of a larva (specimen 5817) preserved in amber from Myanmar in ventral view (p. 397 lower image). The larva was interpreted as representative of Berothidae. Yet, the stylets are slightly curved. Given the overall habitus that resembles that of small larvae of Berothidae, the larva could be a representative of Mantispidae.
(13) Haug et al. [12] provided images of a small larva (specimen 5818) of Mantispidae preserved in amber from Myanmar. Images include micrographs of the entire amber piece with syn-inclusions under cross-polarised light (their Figure 1A p. 3) and ring light illumination (their Figure 1B p. 3), a colour-marked image indicating the position of the larva (their Figure 1C p. 3), the larva in ventral view under ring-light illumination (their Figure 1D p. 3) and under cross-polarised light (their Figure 2A p. 4), a colour-marked image indicating the major morphological features (their Figure 2A p. 4), and a restoration drawing (their Figure 3A p. 5). According to the text, the larva was 0.75 mm long. The specimen was part of the collection of Patrick Müller and is now part of the collection of the Staatliches Museum für Naturkunde, Stuttgart, Germany (SMNS-P-Bu-338). They also re-figured (their Figure 3F p. 5) the larva from Janzen [140].
(14) Pérez-de la Fuente et al. [21] provided images of three larvae preserved in Cretaceous amber from Spain. The first larva (specimen 5819) is of Berothidae. The images include a micrograph of the habitus in ventral view (their Figure 2A p. 5), a drawing of the habitus (their Figure 2B p. 5), a micrograph of the head (their Figure 2C p. 5), a drawing of the head (their Figure 2D p. 5), micrographs of the entire larva in lateral view (their Figure 3A p. 6), of the head in dorsal (their Figure 3B p. 6), ventral (their Figure 3C p. 6), and lateral view (their Figure 3D p. 6), and a detail of the tip of a trunk appendage (their Figure 3E p. 6). According to the provided scale, the larva was about 2 mm long. The specimen is part of the collection Museo de Ciencias Naturales de Álava, Vitoria-Gasteiz, Spain (MCNA 9294).
  The second larva was a possible larva of Berothidae (specimen 5820). Images include a micrograph of the habitus in dorsal view (their Figure 4A p. 7), a drawing of the habitus in dorsal view (their Figure 4B p. 7), and close-up micrographs of the trunk end (their Figure 4C p. 7) and of the head (their Figure 4D p. 7). According to the scale provided, the specimen was about 3.3 mm long. The specimen is part of the collection Fundación Conjunto Paleontológico de Teruel-Dinópolis, Teruel, Spain (SJ-10-25). Unfortunately, the stylets are not fully preserved inside the amber. Therefore, the specimen could not be further considered here.
  The third larva (specimen 5821) could not be easily interpreted in a phylogenetic frame [21], but may be closely related to Berothidae or Mantispidae (“Mantispoidea”) or to Dilaridae (“Dilaridoidea”). The latter was also compared to certain aspects of Osmylidae, but was considered as a less likely interpretation [21]. Images include micrographs in dorso-lateral view from both sides (their Figure 5A,B p. 9), a close-up on trunk sclerites (their Figure 5C p. 9), eye region (their Figure 5D p. 9), a close-up on the latero-ventral part of the head capsule (their Figure 5E p. 9), a close-up on the labial palp (their Figure 5F p. 9), drawings of the head capsule in dorso-lateral view (their Figure 6A p. 10), and the labial palp (their Figure 6B p. 10). According to the provided scale, the head capsule of the specimen was 0.6 mm long. The specimen is part of the collection Fundación Conjunto Paleontológico de Teruel-Dinópolis, Teruel, Spain (SJNB2012-04). As the head is not well accessible in dorsal view, the specimen could not be further considered for our analysis.
(15) Haug et al. [7] reported a larva (specimen 5901) with very long, almost straight stylets, preserved in Myanmar amber: the supersting larva. This larva clearly differs from all others considered here. It is included in this comparison here due to certain similarities of this larva to those of lance lacewings.
  Images included a number of micrographs of the entire amber piece (their Figure 1A p.3), the habitus in ventral (their Figure 1B p. 3) and dorsal view (their Figure 1C p. 3), the head in ventral view (their Figure 1D p. 3), the head capsule in dorsal view under fluorescence light (their Figure 1E p. 3) with colour markings (their Figure 1F p. 3), the head capsule in ventral view (their Figure 1G p. 3), the abdomen in dorsal view (their Figure 1H p. 3), fluorescence images of the entire larva in ventral (their Figure 2A p. 6) and dorsal view (their Figure 2B p. 6), head capsule (their Figure 2D p. 6) and trunk (their Figure 2G p. 6), stereo anaglyphs of the entire larva in dorsal view (their Figure 2C p. 6), the head in ventral view (their Figure 2E p. 3), and a micrograph of the trunk appendages (their Figure 2F p. 6). A simplified restoration of the head in dorsal view (their Figure 4 p. 10) and of the entire specimen in dorsal view (their Figure 5D p. 11) were provided. According to the text, the larva was 2.53 mm long. The specimen was part of the collection of Patrick Müller and is now part of the collection of the Staatliches Museum für Naturkunde, Stuttgart, Germany (SMNS Bu-355).
(16) Haug et al. [146] reported a second specimen of the supersting-type larva (specimen 5902). Most likely, it represents a later developmental stage.

### 3.6. New Fossil Lacewing Larvae with Straight or Almost Straight Stylets

In total, 41 new fossil specimens of lacewing larvae with straight or almost straight stylets (specimens 5822–5862) are described here. These are preserved in 29 amber pieces, which are listed in the following.

(1) Specimen 5822 (Gröhn 7512). A small-sized specimen preserved in a piece of Baltic amber. The larva is accessible in dorsal view (Figure 3), but partly concealed by a thin white film (Verlumung). The larva has very prominent and elongate stylets and is most likely a representative of the group Osmylidae. The larva is 1.66 mm long.
(2) Specimens 5823 + 5824 (BUB 3064). The amber piece includes two specimens, which are preserved in Myanmar amber (Figure 4). Specimen 1 (5823; Figure 4A–C) is well accessible in dorsal view (Figure 4A,B), but partly concealed by an air bubble. The larva has straight stylets. Each trunk appendage bears a prominent attachment structure distally (empodium; Figure 4C). The larva is about 4.8 mm long. Specimen 2 (5824; Figure 4D,E) is well accessible in lateral view and also has straight stylets. The larva is about 7.5 mm long.
(3) Specimen 5825 (BUB 3065). The larva is preserved in Myanmar amber and is well accessible in dorsal view (Figure 5A,B). The head and the straight stylets are well accessible (Figure 5C). Each trunk appendage bears a prominent attachment structure distally (empodium; Figure 5D). The larva is 7.7 mm long.
(4) Specimen 5826 (BUB 3144). The larva is preserved in Myanmar amber. It is well accessible in dorsal view (Figure 6A,B) with the head and straight stylets well visible (Figure 6C,D). The trunk region bears dark patches (Figure 6E). The larva is 3.5 mm long.
(5) Specimen 5827 (BUB 3355). The larva is preserved in Myanmar amber. It is well accessible in dorsal (Figure 7A,B) and ventral view (Figure 7C). In ventral view, the trunk is concealed by a large bubble. The head and the straight stylets are well accessible (Figure 7D,E). Each trunk appendage bears a prominent attachment structure distally (empodium; Figure 7F). The larva is 2 mm long.
(6) Specimen 5828 (BUB 3390). The larva is preserved in Myanmar amber. It is accessible, more or less, in dorsal view (Figure 7G) and ventral view (Figure 7H,I), but partly concealed by dirt particles. The head and the straight stylets are well accessible (Figure 7J,K). The larva is 2.7 mm long.
(7) Specimen 5829 (BUB 3726). The larva is preserved in Myanmar amber. It is well accessible in dorsal view (Figure 8A,B) and partly concealed by a large bubble in ventral view (Figure 8C). The head and the straight stylets are well accessible in dorsal view (Figure 8D,E). Each trunk appendage bears a prominent attachment structure distally (empodium; Figure 8F). The posterior part of the trunk is better accessible in ventral view (Figure 8G). The larva is about 1.1 mm long.
(8) Specimen 5830 (BUB 3741). The larva is preserved in Myanmar amber. It is well accessible in dorsal (Figure 9A,B) and ventral view (Figure 9C), but partly concealed by dirt particles and bubbles. The posterior part of trunk is missing. The head and the straight stylets are well accessible (Figure 9D,E). Each trunk appendage bears a prominent attachment structure distally (empodium; Figure 9F). The larva is 1.4 mm long.
(9) Specimen 5831 (BUB 3962). The larva is preserved in Myanmar amber. It is well accessible in dorsal (Figure 10A,B) and ventral view (Figure 10C). The head and the straight stylets are well accessible (Figure 10D,E). Each trunk appendage bears a prominent attachment structure distally (empodium; Figure 10F). The larva is about 1.7 mm long.
(10) Specimen 5832 (BUB 3963). The larva is preserved in Myanmar amber. It is accessible in ventral (Figure 11A,B) and dorsal view (Figure 11C). The head and the straight stylets are well accessible (Figure 11D,E). Each trunk appendage bears a prominent attachment structure distally (empodium; Figure 11F). The larva is 1 mm long.
(11) Specimen 5833 (CJW F 3197). The larva is preserved in Myanmar amber. It is well accessible in lateral view (Figure 12A,B). The head and the straight stylets are well accessible in latero-dorsal view (Figure 12C). Each trunk appendage bears a prominent attachment structure distally (empodium; Figure 12D). The larva is about 4 mm long.
(12) Specimen 5834 (PED 0380). The larva is preserved in Myanmar amber. It is well accessible in dorsal (Figure 13A,B) and ventral view (Figure 13C). Head, stylets and legs are well accessible (Figure 13D), the stylets are straight. The larva is 1.7 mm long.
(13) Specimen 5835 (CJW F 3198). The larva is preserved in Myanmar amber. It is well accessible in dorsal view (Figure 13E,F). The head and the straight stylets are well accessible (Figure 13G). The posterior part of the trunk is well accessible (Figure 13H). The larva is 1.1 mm long.
(14) Specimen 5836 (CJW F 3336). The larva is preserved in Myanmar amber. It is accessible in dorsal view (Figure 13I,J), but the abdomen is flipped over. The stylets are straight. The larva is about 0.9 mm long.
(15) Specimens 5837 + 5838 (PED 0772). The amber piece includes two specimens, which are preserved in Myanmar amber (Figure 14). Specimen 1 (5837; Figure 14A–E) is accessible in dorsal (Figure 14A,B) and ventral view (Figure 14C), but partly concealed by bubbles on both sides. The head and the straight stylets are accessible in dorsal view (Figure 14D,E). The larva is 1.1 mm long.
  Specimen 2 (5838; Figure 14F–H) is accessible in dorsal (Figure 14F,G) and ventral view (Figure 14H), but strongly concealed by bubbles on both sides. The stylets are straight. The larva is 1.1 mm long.
(16) Specimen 5839 (PED 0828). The larva is preserved in Myanmar amber. It is well accessible in ventral (Figure 15A,B) and dorsal view (Figure 15C). The head and the straight stylets are well accessible (Figure 15D,E). Each trunk appendage bears a prominent attachment structure distally (empodium; Figure 15F). The larva is 2.1 mm long.
(17) Specimens 5840–5846 (PED 0900). The amber piece includes seven specimens, which are preserved in Myanmar amber (Figure 16, Figure 17 and Figure 18). Specimen 1 (5840; Figure 16A–E) is well accessible in dorsal (Figure 16A,B) and ventral view (Figure 16C), but partly concealed. The head and the straight stylets are well accessible (Figure 16D,E). The larva is 1.3 mm long.
  Specimen 2 (5841; Figure 16F–I) is well accessible in dorsal (Figure 16F,G) and ventral view (Figure 16H). The stylets are straight. Each trunk appendage bears a prominent attachment structure distally (empodium; Figure 16I). The larva is 1.1 mm long.
  Specimen 3 (5842; Figure 17A–E) is well accessible in dorsal (Figure 17A,B) and ventral view (Figure 17C), but partly concealed by Verlumung. The head and the straight stylets are well accessible (Figure 17D,E). The larva is 1.2 mm long.
  Specimen 4 (5843; Figure 17F–I) is well accessible in dorsal view (Figure 17F,G), but the posterior part of the abdomen is missing. The head and the straight stylets are well accessible (Figure 17H,I). The larva is 0.8 mm long.
  Specimen 5 and 6 (5844 and 5845; Figure 18A,B) are lying on top of each other. They are well accessible in dorsal view (Figure 18A,B), but partly concealed by dirt particles. The stylets are straight. Each of the two larvae is about 1 mm long.
  Specimen 7 (5846; Figure 18C–F) is well accessible in ventral view (Figure 18C,D), but partly concealed by dirt particles. The head and the straight stylets are well accessible (Figure 18E,F). The larva is 1.2 mm long.
(18) Specimens 5847 + 5848 (PED 0769). The amber piece includes two specimens, which are preserved in Myanmar amber (Figure 19). Specimen 1 (5847; Figure 19A–E) is well accessible in dorsal view (Figure 19A,B). The head and the straight stylets are well accessible (Figure 19C,D). Each trunk appendage bears a prominent attachment structure distally (empodium; Figure 19E). The larva is 1.3 mm long.
  Specimen 2 (5848; Figure 19F–I) is well accessible in ventral view (Figure 19F,G). Head and stylets are well accessible (Figure 19H,I), the stylets are slightly curved inwards. The larva is 1.4 mm long.
(19) Specimen 5849 (BUB 3711a). The larva is preserved in Myanmar amber. It is well accessible in latero-ventral (Figure 20A) and latero-dorsal view (Figure 20B,C), but partly concealed by bubbles. The stylets are slightly curved inwards. The larva is about 2.4 mm long.
(20) Specimens 5850–5853 (PED 0791). The amber piece includes four specimens, which are preserved in Myanmar amber (Figure 21 and Figure 22). Specimen 1 (5850; Figure 21A–E) is well accessible in ventral (Figure 21A,B) and dorsal view (Figure 21C). Head and stylets are well accessible (Figure 21D,E), and the stylets are slightly curved inwards. The larva is 1.4 mm long.
  Specimen 2 (5851; Figure 21F–H) is well accessible in ventral (Figure 21F,G) and dorsal view (Figure 21H). The stylets are slightly curved inwards. The larva is 1 mm long.
  Specimen 3 (5852; Figure 22A–C) is accessible in ventral (Figure 22A,B) and dorsal view (Figure 22C), but partly destroyed. The stylets are slightly curved inwards. The larva is about 2.2 mm long.
  Specimen 4 (5853; Figure 22D–H) is well accessible in ventral (Figure 22D,E) and dorsal view (Figure 22F). The head is separated from the body. Head and stylets are well accessible (Figure 22G,H), and the stylets are slightly curved inward.
(21) Specimen 5854 (PED 0823). The larva is preserved in Myanmar amber. It is well accessible in ventral (Figure 23A,B) and dorsal view (Figure 23C), but partly concealed by dirt particles. Head and stylets are well accessible (Figure 23D,E), and the stylets are slightly curved inwards. Each trunk appendage bears a prominent attachment structure distally (empodium; Figure 23F). The larva is 1.4 mm long.
(22) Specimen 5855 (PED 0898). The larva is preserved in Myanmar amber. It is well accessible in dorsal (Figure 24A,B) and ventral view (Figure 24C). The stylets are slightly curved inwards. The larva is 1.4 mm long.
(23) Specimen 5856 (PED 0899). The larva is preserved in Myanmar amber. It is well accessible in dorsal (Figure 25A,B) and ventral view (Figure 25C), but partly concealed by bubbles. Head and stylets are well accessible (Figure 25D,E), and the stylets are slightly curved inwards. Each trunk appendage bears a prominent attachment structure distally (empodium; Figure 25F). The larva is 1.4 mm long.
(24) Specimen 5857 (BUB 0049). The larva is preserved in Myanmar amber. It is well accessible in ventral view (Figure 26A,B), but small areas of the trunk are partly concealed by a bubble. Head and stylets are well accessible (Figure 26C,D), and the stylets are elongated and slightly curved outwards. Each trunk segment bears a pair of prominent setae on each side (Figure 26E). Each trunk appendage bears a prominent attachment structure distally (empodium, Figure 26F). The larva is 4 mm long.
(25) Specimen 5858 (BUB 3368). The larva is preserved in Myanmar amber. It is well accessible in dorsal view (Figure 27A,B), but partly concealed by dirt particles. Head and stylets are well accessible (Figure 27C), and the stylets are elongated and slightly curved outwards. The larva is about 3.5 mm long.
(26) Specimen 5859 (BUB 3737). The larva is preserved in Myanmar amber. It is accessible in dorsal (Figure 28A,B) and ventral view (Figure 28C), but partly concealed by dirt particles. Head and stylets are accessible (Figure 28D,E), and the stylets are elongated and slightly curved outwards. The larva is about 2.2 mm long.
(27) Specimen 5860 (PED 0587). The larva is preserved in Myanmar amber. It is well accessible in dorsal (Figure 29A,B) and ventral view (Figure 29C), but the terminal end is partly concealed by a bubble. The antennae bear prominent setae distally (Figure 29D). The larva is 4.5 mm long.
(28) Specimen 5861 (PED 0627). The larva is preserved in Myanmar amber. It is accessible in dorsal (Figure 30A,B) and ventral view (Figure 30C), but partly concealed by dirt particles in dorsal view and strongly concealed by bubbles and dirt particles in ventral view. The stylets are elongate and slightly curved outwards. The larva is about 2.8 mm long.
(29) Specimen 5862 (PED 0790). The larva is preserved in Myanmar amber. It is accessible in dorso-lateral (Figure 31A,B) and ventro-lateral view (Figure 31C), but partly concealed by dirt particles. The stylets are elongated and slightly curved outwards. The larva is about 2.1 mm long.

### 3.7. Results of the Shape Analysis

Regarding the extant larvae, the data set contains 12 larvae of Berothidae, 33 larvae of Mantispidae, 9 larvae of Dilaridae, and 28 larvae of Osmylidae, thus 82 extant larvae in total. Of the 64 fossils, 43 specimens were included into the analysis, as in the remaining specimens, the head was either incomplete or not preserved at the right angle.

The analysis resulted in only three effective principal components (Figure S1, Text S1; for details on the analysis, see additional Supplementary Files S1–S5). PC1 explains 84.95% of the overall variation. It was dominated by the relative length of the stylets and the posterior edge of the head capsule. A low value indicates rather short stylets and a straight posterior edge of the head capsule. A high value indicates rather long stylets and a rounded posterior edge of the head capsule.

PC2 explains 8.15% of the overall variation. It was dominated by the relative length of the stylet. A low value indicates rather long stylets, and a high value indicates rather short ones.

PC3 explains 3.54% of the overall variation. It was dominated by the shape of the tip of the stylets. A low values indicates a rather broad tip, and a high value indicates a more pointed tip.

Plotting PC2 versus PC1 resulted in a tilted L-shaped occupied area of the morphospace (Figure 32). Areas occupied by modern representatives overlapped in certain regions. The area occupied by larvae of Berothidae overlapped largely with that of Dilaridae, almost enveloping it entirely. On the right part of the plot, the area occupied by larvae of Osmylidae was separated from that of Berothidae. On the left side of the plot, a large area was occupied by extant larvae of Mantispidae. This area overlapped partly with that of Berothidae.

The area occupied by the fossil larvae largely overlapped with those occupied by the different groups of extant larvae, forming a rather straight line from the lower left to the upper right. The smallest overlap was with the extant larvae of Mantispidae (Figure 33).

## 4. Discussion

### 4.1. Identity of the Fossils

Fossil larvae are often quite challenging to interpret in a taxonomic or phylogenetic frame for a number of reasons. Descriptions and also documentation of details of extant larval specimens often focus on very specific features that are considered to be important for taxonomy. These features are not necessarily accessible in a fossil. The features of modern-day larvae have been identified based on comparisons of several of these, leading to the recognition that they are of taxonomic value. Yet, the fossil larvae may be representatives of outgroups to the extant ones where such characters are not necessarily informative. This can lead to the strange situation in which there are, in fact, features available in the fossil larvae, but there is no real comparable frame available.

Hence, only in cases in which the modern larvae are very well known (including some variability within the group) and the fossils allow access to many small details can we can make a more educated guess or even perform a strict analysis. The latter has so far been possible in very few cases [91,147]. Yet, shape can also be informative to a certain degree. Shape is basically a phenetic type of information; it is influenced by functional needs and phylogenetic background (although these two cannot be fully separated from each other). For most of the fossil larvae treated here, we can therefore compare the shape to that of their extant counterparts and then additionally use qualitative data to further narrow down their identity.

### 4.2. Fossil Representatives of Osmylidae

Specimens 5802, 5808, 5822, and 5857–5860 plotted close to or directly within the area occupied by modern representatives of Osmylidae (Figure 33). Most of these specimens, besides 5858, have clearly outward curved stylets, further supporting the interpretation of the fossils as representatives of Osmylidae. Two of these specimens have already been interpreted in this way (5802 in [138]; 5808 in [142]). No structures were apparent that would contradict this interpretation, with one possible exception that needs to be discussed.

Specimen 5857 possesses prominent empodia. Larvae of Osmylidae are often considered to lack empodia [148]. Yet, at least some of the extant larvae appear to have small empodia [115] (his Figure 1 pl. IX). This implies that the seeming absence of prominent empodia in most lance lacewing larvae might be a derived feature. Specimen 5857 is therefore interpreted as a lance lacewing larva that has retained the plesiomorphic state, i.e., still possessing empodia.

In the same area, the two supersting larvae (specimens 5901 and 5902) were plotted. The similarity of supersting larvae and larvae of Osmylidae has been noted before [7].

### 4.3. Fossil Representatives of Mantispidae

Specimens 5816, 5818, and 5825 plotted within the area occupied by modern representatives of Mantispidae, outside the range occupied by similar appearing larvae, such as Berothidae (Figure 33). Specimen 5818 has been found in direct association with a spider and was already interpreted as a representative of Mantispidae [12].

Specimen 5816 was originally interpreted as a possible larva of Coniopterygidae [144], but later re-interpreted as a larva of Berothidae [21]. The specimen is indeed not easy to interpret. This is caused, among other factors, by the asymmetric appearing stylets. The stylet seen on the left side in ventral view [144] (his Figure 6) is slightly curved, and the right stylet appears straight. We decided to use the left stylet for analysis as the right stylet appears shorter, hence being apparently broken off and lacking the distal part. A slightly curved stylet is also more compatible with an interpretation as a larva of Mantispidae. The rather short and broad head is also more characteristic for larvae of Mantispidae.

The morphology of the antennae is even more problematic as it does not appear to be club-shaped as in many modern mantis lacewing larvae, but rather slender as in larvae of Berothidae. We still consider this larva as a possible representative of Mantispidae, yet the case is significantly weaker than in the case of specimen 5818.

Specimen 5825 is also difficult to interpret and quite small. In this case as well, the rather broad and short head is well compatible with an interpretation as a larva of Mantispidae. In this specimen, the antennae and palps additionally have slightly bulging elements instead of simple tube-shaped ones. This further supports an interpretation of this larva as a mantis lacewing. 

### 4.4. Fossil Representatives of Berothidae and Dilaridae

The majority of the fossil larvae plot in the area occupied by modern larval representatives of Berothidae, but also Dilaridae (Figure 33). Most of the fossils show similarities to modern-day larvae of Berothidae. Specimen 5828 has a particularly elongated head capsule and relatively long stylets. In these aspects, the specimen resembles some of the modern larvae of Dilaridae (e.g., 5402, 5406). It is therefore a good candidate for representing a fossil larva of Dilaridae.

In most other specimens, the case is much less clear. It appears that the morphology of larvae of Berothidae is characterised by numerous plesiomorphies retained from the ground pattern of Mantispoidea. It is therefore not easily possible to clearly identify the fossils as representatives of Berothidae, as the principle morphology is likely already present in the early lineage of Mantispoidea (including Mantispidae, Berothidae and Rhachiberothidae, possibly an ingroup of the latter).

The overlapping area between the two groups Berothidae and Dilaridae complicates the situation. In recent phylogenetic analyses [149], Mantispoidea and Dilaridae are not sister groups, indicating that the similar appearing morphology of the larvae evolved at least twice independently. The fact that only a few extant larvae of the two groups are known is also problematic in this aspect. The variability of the larval morphology in the two groups remains therefore effectively largely unknown.

For the younger fossils from the Eocene, the morphology of the larvae is well accessible, providing important details. Hence, for these, an interpretation as larvae of Berothidae is well supported [13].

For many of the Cretaceous fossils the case is more complicated. Many of these remain good candidates for representing larvae of Berothidae. Yet, these may as well also be larvae of the representatives of the early lineage of Mantispoidea, or also of the group Dilaridae.

### 4.5. The More Problematic Fossils

Specimens 5817, 5848, 5850, 5851, 5853, 5854, and 5856 are more problematic to interpret. Many of these (5817, 5848, 5850, 5851, 5853) plotted close to or within the area occupied by extant larvae of Berothidae and Dilaridae (Figure 33). Yet, they differ significantly from larvae of Berothidae and Dilaridae in several aspects. First, and most prominently, the stylets are curved.

The main reason to include these larvae into the present analysis is the morphology of the antennae and labial palps, which are club-like. Such a morphology is also present in larvae of Mantispidae; within Mantispidae curved stylets are also known (e.g., specimens 5205, 5213, 5214, 5217, 5218). Hence, our original expectation was that the problematic fossil specimens could plot close to modern larvae of Mantispidae. Yet, this was not the case.

Comparing the fossils to other larvae of the group Neuroptera, one might also suggest that these larvae are in fact representatives of the group Hemerobiidae. Extant larvae of this group also have club-like labial palps [150] (their Figure 1B) [151] (their Figure 1) [152] (their Figure 1) [153] (his Figure 19a,d). Yet, they do not possess club-like antennae, but rather slender ones.

Hence, these fossil larvae remain problematic to interpret as they share similarities with modern larvae of Hemerobiidae, but also with Mantispidae. We have not yet explored the overall morphological diversity of Hemerobiidae and can therefore not compare the shape of these.

Yet, the character distribution provides a certain signal. The broadened labial palps in extant larvae of Hemerobiidae and Mantispidae are clearly a case of convergence. While it might be argued that the broadened palps in the fossils are another case of convergence, it seems more likely that they are either closer related to Hemerobiidae or to Mantispidae. The same is true for the broadened antennae. While one could argue that the broadened antennae in the fossils and in Mantispidae are a case of convergence, it is more parsimonious to interpret this as a shared derived character. The difference in shape of the fossils and the modern larvae can, in this case, be simply interpreted as a plesiomorphy in the fossils. The fossils plot in the area where modern larvae of Berothidae plot. As pointed out above, we can assume that the principle shape of the heads in larvae of Berothidae is potentially the plesiomorphic condition for Mantispoidea.

We therefore suggest that the fossils represent a more ancestral larval morphology of Mantispidae, retaining a plesiomorphic shape, but already possessing slightly curved stylets and club-like antennae and palps. This morphology is no longer present in the modern fauna. A future quantitative comparison including Hemerobiidae may further corroborate this interpretation.

### 4.6. Diversity of Berothidae and Dilaridae over Time

The group Berothidae is not very species-rich, with only about 100 species in the modern fauna and about 35 known extinct species based on adult specimens [154]. A correlation between fossil larvae and adults of lacewings is largely hampered by the lack of fossil pupae (but see [155]). Concerning the morphological diversity of the larvae of Berothidae, there seems to have been minor changes over time in this aspect; 100 million years ago, larvae that resemble modern-day larvae of Berothidae were already present. The overall occupied shape space seems not to have changed significantly, indicating no change of the morphological diversity (Figure 33).

Wedmann et al. [13] discussed the possible association of larvae of Berothidae with termites, as seen in the modern fauna, for larvae from the Eocene. This association might account for the specimens from the Cretaceous. Yet, we need to consider that the fossil larvae, especially those from the Cretaceous, are not necessarily ingroup representatives of Berothidae, or at least not representatives of the modern lineages that interact with termites. We can therefore not use the argument of phylogenetic bracketing [156] in this case. Furthermore, we need to consider preservational aspects. If these larvae would have lived inside termite nests, the preservation potential of such larvae in amber should be rather low. This does not exclude that larvae potentially and occasionally entered termite nests but also spent time outside to become trapped in the tree resin.

Dilaridae seems about as species-rich as Berothidae in the modern fauna, yet clearly fewer larvae of Dilaridae are known. This is not only true for the extant fauna, as very few possible fossil larvae of Dilaridae are known. The only possible specimen in the literature was reported by Pérez-de la Fuente et al. [21]; yet, the available characters were inconclusive and would also fit with an interpretation of the larva as Mantispoidea.

As pointed out, specimen 5828 is a good candidate for representing a larva of Dilaridae. Yet, with a single fossil specimen there is no basis for comparing morphological diversity. The few known adult fossils of Dilaridae indicate a higher morphological diversity of these in the past as exemplified by specialised mouthparts not known from modern adults [157].

### 4.7. Diversity of Osmylidae over Time

The fossil record of adults of Osmylidae was recently summarised in Winterton et al. [10]. Many modern larvae of Osmylidae are semi-aquatic [129], while some are also fully terrestrial [23,133]. This makes the interpretation of the life style of fossil larvae of this group challenging, at best. An aquatic life style might lower the preservation potential, yet in fact there are numerous examples of aquatic and even marine organisms preserved in various types of amber [142,158,159,160,161].

Still, quite few fossil larvae of Osmylidae have been reported so far. The smaller area of shape space occupied by fossils compared with the extant representatives (Figure 33) may be caused by a simple sampling bias. Yet, clearly all fossils fall well into the modern range. Even the two supersting fossils do not expand from this range. An interesting point to note is the absence of larvae of Osmylidae as compression fossils. At least for the semi-aquatic forms, one should expect such fossils, yet so far none have been reported [162].

### 4.8. Diversity of Mantispidae over Time

Extant larvae of Mantispidae spread over quite a wide range of the shape space. Of the fossils, only three larvae plot in the area of the modern forms, but still closely to the area occupied by Berothidae (Figure 33; likely representing the plesiomorphic condition). Other fossil larvae that may be representatives of Mantispidae plot outside the area occupied by modern larvae.

Adult forms of Mantispidae are well known from Cretaceous ambers [163,164,165,166,167]. These fossils are apparently representatives of early diverging branches of Mantispidae [165,166]. Hence, Mantispidae seems to have undergone an early diversification already in the Cretaceous. The fossil larvae with curved stylets may therefore be the larval stages of these early forms. Some of the fossil adults possess morphologies that are no longer present in modern forms. It should therefore not be surprising to see a similar pattern in the fossil larvae with larval morphologies that are now extinct.

Hence, the modern fauna seems to have lost some morphological diversity present in the Cretaceous. On the contrary, the modern fauna includes quite a larger morphological diversity of larvae of Mantispidae. These diverse forms have likely evolved only after the Cretaceous. Although the sample size of the Cretaceous larvae is much smaller than that of the modern forms, the difference is so strongly expressed that it cannot be easily explained by a sampling bias. Hence, the overall morphological diversity of larvae of Mantispidae seems to have increased significantly over time. Part of the difference between modern and Cretaceous fauna may be attributed to the lack of clear stage 2 and 3 larvae in the Cretaceous. It remains unclear whether this absence simply means that we have not yet found such a larva, or that this morphology had simply not yet evolved (see also discussion in [12]).

It may sound counter-intuitive to observe a loss of morphological diversity in adult mantis lacewings, while at the same time the morphological diversity of the larvae has significantly increased. Yet, this only demonstrates that, especially in representatives of Holometabola, there is a certain independence of the ecology of the adults and the larvae. This observation also emphasises that diversity, in the sense of ecological diversity or ecosystem function diversity, is not easily represented by simply counting species.

For the more modern-appearing larvae of Mantispidae, we can assume a similar life style to many modern larvae, with a parasitic interaction with web-spinning spiders (see discussion in [12]). We cannot assume that for the supposedly early larvae with curved stylets. As these larvae possibly represent early branches, we can not make use of phylogenetic bracketing here [156]. Consequently, it is not easily possible to speculate about the life habits of these larvae. They may still have lived as predators.

### 4.9. Diversity of Lacewing Larvae over Time

The group Neuroptera has been generally assumed to have been more diverse in the past. Indeed, the Cretaceous has revealed an astonishing morphological diversity of lacewing larvae [7,18,21,91,147,155,164,168,169,170,171,172,173]. Quantitative morphological comparisons of several lineages have revealed a distinct loss of larval morphological diversity in silky lacewings (Psychopsidae) and split-footed lacewings (Nymphidae), as well as a shift in thread-winged lacewings (Crocinae) and an indecisive pattern due to sample size problems in spoon-winged lacewings (Nemopterinae). We can now add Berothidae without a significant shift, Dilaridae with an indecisive pattern (due to sample size), Osmylidae without significant change (yet less clearly, also due to sample size), and Mantispidae, which lost a certain part of the morphospace, but saw a significant increase of diversity towards the modern fauna.

Hence, the pattern is apparently more complex than a simple overall loss in all lineages. Instead, there was a loss in some lineages, a more or less constant diversity in others, but in at least one lineage also a distinct increase of morphological diversity. The observation that the increase of morphological diversity in larval mantis lacewings is coupled to a loss of adult diversity further demonstrates that changes in diversity over time are more complex than often anticipated.

## Data Availability

All data from this study are available in this paper and the associated Supplementary Materials.

## Supplementary Materials

The following are available online at https://zenodo.org/record/5558408#.YWFb-vkzaUk, Figure S1: Graphical representation of the factor loadings of the shape analysis of the heads, Table S1: Detail information on all specimens included in this study with their geological occurrence (note that no specimen is from Miocene despite large Miocene amber abundances, abbreviations: K = Cretaceous, Eoc = Eocene, Mio = Miocene, PC = principal component), Text S1: Results of the principal component analysis of the head shapes, Supplementary files S1–S5: Files resulting from the shape analysis, including chain codes, aligned shapes, and principal component analysis. Figures 1–33 of high resolution are available in the supplementary materials.
